# Factors Associated with Retention in Routine Well-Care Visits Among Children of Adolescent Mothers Living With and Without HIV: A Community-Based Study in the Eastern Cape, South Africa

**DOI:** 10.1136/bmjopen-2025-106412

**Published:** 2025-12-31

**Authors:** Camille Wittesaele, Elona Toska, Lucie D. Cluver, Helen A. Weiss, Aoife M. Doyle

**Affiliations:** 1 International Statistics and Epidemiology Group, London School of Hygiene & Tropical Medicine, London, UK; 2 Department of Social Policy and Intervention, University of Oxford, Oxford, UK; 3 Centre for Social Science Research, University of Cape Town, Rondebosch, South Africa; 4 Department of Public Health, Institute of Tropical Medicine Antwerp, Antwerp, Belgium; 5 Department of Psychiatry and Mental Health, University of Cape Town, Rondebosch, South Africa; 6 Biomedical Research and Training Institute, Harare, Zimbabwe

**Keywords:** Health Services Accessibility, Primary Health Care, Person-Centered Care, Community child health, Preventive Health Services

## Abstract

**Abstract:**

**Objective:**

To describe well-care visit attendance among children of adolescent mothers living with HIV and HIV-negative adolescent mothers and identify factors associated with optimal retention in the well-care visit schedule up to 18 months.

**Design, setting, participants:**

Cross-sectional data were used from a community-based observational cohort study of adolescent mothers (10–19 years; n=481) and their children (≥19 months old; n=502) in the Eastern Cape, South Africa.

**Outcome:**

Optimal well-care visit retention up to 18 months was defined as attending visits within 4 weeks of the recommended child age, attending the 18-month visit and missing no more than one scheduled visit.

**Results:**

Attendance was highest at the 6-week visit (88.4%; 95% confidence interval (CI) 85.6% to 91.3%) and lowest at the 18-month visit (58.0%, 95% CI 53.6% to 62.3%). About one-third (36.1%; 95% CI 31.8% to 40.3%) of children were retained to 18 months. Retention was highest among children living in rural vs urban areas (adjusted odds ratio (aOR)=2.01, 95% CI 1.32 to 3.06), those born to mothers whose highest education at pregnancy was secondary versus primary school (aOR=2.73, 95% CI 1.60 to 4.65), born via caesarean section vs vaginal birth (aOR=1.65, 95% CI 1.05 to 2.60) and living closer to the clinic (aOR=0.52, 95% CI 0.28 to 0.96 for long vs short distance). There was weak evidence that retention was lower among children of mothers living with HIV (aOR=0.64, 95% CI 0.40 to 1.02) and higher among food-secure children (aOR=2.18, 95% CI 0.96 to 4.96) and those receiving the child support grant (aOR=1.71, 95% CI 0.92 to 3.16).

**Conclusions:**

Universal interventions are needed for retention beyond the neonatal period for children of adolescent mothers living with HIV and HIV-negative adolescent mothers. Interventions must address structural barriers, especially for adolescent mothers with primary education and in urban areas. Future research should examine the underlying mechanisms linking mode of delivery with well-care retention.

**STRENGTHS AND LIMITATIONS OF THIS STUDY**

- Statistical analyses were informed by a conceptual framework developed using insights from qualitative interviews with adolescent mothers among the cohort.
- Health facility-based and community-based sampling reduced selection bias and improved the generalisability of findings to children of adolescent mothers in rural and urban settings, who may or may not have had access to health services at the time of recruitment.
- Using both vaccination and well-care visit attendance data improved accuracy in measuring attendance and helped distinguish true missed visits from those due to vaccine stock-outs or incomplete records.
- Factors including maternal knowledge of well-care services and vaccine stock-outs could explain variations in retention but were not measured in this study.
- The cross-sectional measurement of time-varying factors, including maternal return to school after giving birth and cohabitation with the child, limited our ability to assess how changes over time may influence retention in the well-care visit schedule.

## Background

 Global declines in child mortality and morbidity in the last three decades have been attributed to the delivery of health promotion and prevention services.^1 2^ However, inequities persist and children born to adolescent mothers (10–19 years old) remain at higher risk of poor outcomes compared with those born to older mothers.^3^ In 2024, the WHO issued guidance for routine well-care visits to bolster the delivery of health promotion and prevention services for children.^4^ In South Africa, recent data show increases in adolescent birth rates, particularly among younger adolescents (10–14 years old).^5^ Compared with children born to adult mothers, children born to adolescent mothers face increased risks of poor birth outcomes,^6^ stunting and undernutrition,^7 8^ delayed cognitive development,^9 10^ reduced breastfeeding^11 12^ and a threefold higher risk of vertical HIV transmission.^13^ It is vital that these children attend well-care visits to monitor their healthy growth and development and to mitigate the long-term impacts of these outcomes.

In line with WHO guidance, each well-care visit in South Africa is timed to deliver age-appropriate health promotion and prevention services (table 1).^14^ Routine attendance and retention in the well-care visit schedule through to the 18-month visit is crucial for assessing children’s health and development and for providing early intervention before visits become less frequent. At 18 months, cognitive and motor skills development accelerates significantly, making it a crucial window for detecting and addressing developmental delays.^15^ In South Africa, this coincides with the last scheduled childhood vaccination and children with perinatal HIV exposure also receive a confirmatory HIV test at this visit—marking their exit from the Prevention of Mother-to-Child Transmission of HIV (PMTCT) programme.

**Table 1:** Schematic overview of WHO and South African well-care visit schedules, services delivered and attendance age thresholds

Child age	Well-care visit schedule		Well-care services‡	Visits assessed	Age threshold for defining attendance§
	WHO*	South Africa†			
Day 3–6	•	•	A, B, D, E	•	2–31 days
2 weeks	•				
6 weeks	•	•	A, B, D, E	•	5–8 weeks
7 weeks					
8 weeks					
9 weeks					
10 weeks	•	•	A, B, D, E	•	9–12 weeks
11 weeks					
12 weeks					
13 weeks					
14 weeks	•	•	A, D, E	•	13–16 weeks
4 months		•	A, C, E		
5 months		•	A, C, E		
6 months	•	•	A, B, C, D¶, E, F, H	•	25–33 weeks
7 months		•	A, C		
8 months		•	A, C		
9 months	•	•	A, C, D, H	•	38–47 weeks
10 months		•	A, C		
11 months		•	A, C		
12 months	•	•	A, B, C, D¶, F, G, I	•	51–59 weeks
13 months					
14 months		•	A, C		
15 months					
16 months		•	A, C		
17 months					
18 months	•	•	A, B, C, D, F, G, H	•	77–86 weeks

* Summarised from WHO guidance scheduled child and adolescent well-care visits.^4^

† Based on Road to Health Booklet (version issued in 2010).

‡ A=growth monitoring; B=PMTCT/HIV; C=tuberculosis screening; D=immunisations; E=feeding counselling; F=vitamin A; G=deworming; H=development screening; I=oral health screening.

§ Within 4 weeks of recommended age.

¶ In December 2015, the National Expanded Programme on Immunisation revised its schedule, moving the first two doses of the measles-containing vaccine to 6 and 12 months*, *thereby adding two additional vaccination visits.

PMTCT, Prevention of Mother-to-Child Transmission.

Adolescent girls and mothers have an unmet need for sexual and reproductive health services,^16^ face barriers to healthcare access,^17 18^ lower retention in HIV-related care and PMTCT programmes,^13 19^ and experience stigma from healthcare workers.^20^,^22^ Coverage of services delivered at well-care visits decreases as their children age.^23^,^25^ In a recent community-based cohort study of children born to adolescent mothers in the Eastern Cape Province (2017–2019), we found a decline in vaccine coverage and timeliness along the schedule,^23^ and fewer than half of children attended the 18-month well-care visit by 19 months old.^26^

Research on risk factors for missed follow-up visits in Eastern and Southern Africa has focused on postnatal care attendance during the neonatal period (i.e., <1 month old)^27 28^ or retention in PMTCT programmes.^19^ Little is known about factors that are associated with retention in well-care visits beyond the neonatal period, particularly for children without perinatal HIV exposure and those born to adolescent mothers. In South Africa, a nationally representative cohort study (2010–2014) of mother–infant pairs (n=27 699 mothers; mean age 26 years) found that adolescent mothers (≤19 years) were less likely than older mothers to attend at least three postnatal visits within 6 weeks post partum^27^ and were more likely to miss postpartum follow-up visits, including the 18-month visit.^29^ Nearly one-third of children in this study missed their 18-month visit, though this may be underestimated due to research participation incentives.

Qualitative research in South Africa has identified barriers faced by adolescent and adult caregivers accessing growth monitoring services at well-care visits. Adult caregivers in Limpopo Province valued growth monitoring and knowledge of the importance these services were crucial for ongoing attendance.^30^,^32^ Barriers such as work commitments, transportation costs, long wait times and poor service quality (e.g., vaccine stock-outs, negative attitudes from healthcare workers) deterred attendance.^31^ In the Eastern Cape, adolescent mothers faced additional challenges like school commitments despite strong motivation to attend visits.^26^

Quantitative evidence on retention in well-care services for children of adolescent mothers, both with and without perinatal HIV exposure, remains limited. Identifying factors that are associated with retention is crucial to support access to essential child health services, particularly for children of adolescent mothers, who face increased risks of adverse health and developmental outcomes. This study describes well-care visit attendance and identifies factors associated with optimal retention through to the 18-month visit among children born to adolescent mothers in the Eastern Cape, South Africa.

## Methods

### Study design, recruitment and data collection

Adolescent and young mothers (10–24 years old) (n=1044) and their children (n=1154) living in the Eastern Cape, South Africa, were recruited into an observational cohort study (March 2017–July 2019).^33^ Adolescent and young mothers were recruited using both health facility-based and community-based approaches, including government healthcare facilities providing HIV services, maternity obstetric units, door-to-door recruitment, secondary schools, non-governmental organisations and social service referrals, peer/self-referral, and other community venues (e.g., hair salons, malls, churches).^33^ All children born to eligible mothers were eligible for enrolment in the cohort study, regardless of their cohabitation arrangements with their biological mother. This sampling strategy, implemented across rural and urban settings, improved representativeness and reduced selection bias towards health service users, addressing a common limitation in prior research that relied solely on health facility-based sampling.^2730^,^32 34^ It enabled inclusion of mothers and children with limited or no health service access and aligns with WHO guidance promoting decentralised, community-based recruitment to enhance representativeness and relevance of research findings.^35^

At baseline, children ranged from 3 weeks to 9 years old and were born between 2009 and 2019. Sociodemographic and healthcare access data were collected from mothers through self-administered surveys on tablets, with support from research assistants. Cross-sectional data (2017–2019) about children’s access to health services were collected from primary caregivers where adolescent mothers were not cohabiting with their children. In South Africa, mothers are issued Road to Health booklets postdelivery which are used to document their child’s vaccination status and well-care visit attendance. Well-care visit and vaccination dates were extracted from photographs of pages for all available Road to Health booklets. A reflexivity statement is included in online supplement 1. Further details about data collection are described elsewhere.^23^

### Sampling

This analysis was restricted to children born to adolescent girls (10–19 years old) with complete baseline survey data and original Road to Health booklets available at baseline (see online supplement 2 for the sampling flow chart). To ensure that well-care visit attendance data up to 18 months was available, only children who were at least 19 months old when their Road to Health booklets were photographed were included. Consequently, only those born before May 2018 were eligible, as they would have reached their 18-month visit by December 2019, with a 4-week window for attendance observation. Complete survey data and original Road to Health booklets were available for 928 children, of whom 542 were at least 19 months old at data collection and had sufficient follow-up well-care follow-up records. Of these, 12 non-original booklets were excluded (i.e., data were missing because the original booklets had been replaced), and 28 were excluded due to extensive damage or missing pages. As a result, 502 children were included in the analysis.

#### Conceptual framework

**Figure 1:**
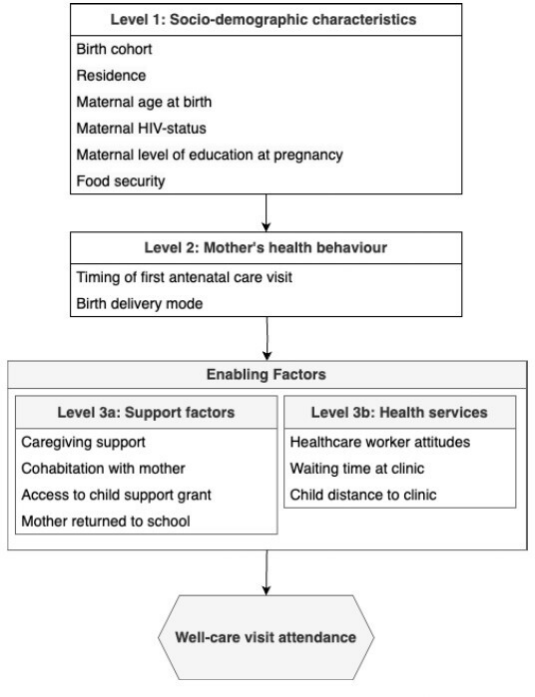
Conceptual hierarchical framework for multivariable analysis of factors associated with well-care visit retention.

We used a hierarchical conceptual framework (figure 1) to guide analyses of the associations between support-related and health service factors and retention in the well-care visit schedule up to 18 months. The framework was developed using insights from a prior exploratory mixed-methods study of well-care visit attendance among children of adolescent mothers^26^ and with reference to relevant literature.^1936^,^38^ The exploratory study measured the proportion of well-care visits attended up to 18 months using data extracted from the Road to Health booklets. Factors influencing attendance were explored using semistructured interviews with adolescent mothers. We found that mothers were motivated to attend well-care services; however, this contradicted a recorded decline in overall attendance. A complex dynamic existed between barriers to accessing health services (e.g., distance to health facilities and long queues at clinics) and support-related factors that facilitated attendance (e.g., caregiving support and access to child support grant).

Given that children are dependent on their mothers to attend well-care visits, the framework includes both child and maternal characteristics. Level 1 includes socioeconomic factors (i.e., food security, maternal level of education) and demographic characteristics (i.e., child sex, child’s birth cohort, urban/rural residence, maternal age at birth and maternal HIV status). Birth cohort was included to account for the 2015 update to South Africa’s Expanded Programme on Immunisation (EPI), which added vaccinations to two existing well-care visits.^39^ This may have prompted more consistent attendance among children born after the vaccination schedule change. Children were categorised as being born before (i.e., EPI 2009) or after (i.e., EPI 2015) the programme update. Food security was defined as experiencing at least 1 day without enough food at home in the past week and was included as a proxy for household income.^40^ Maternal HIV status was recorded only at the time of data collection and verified using self-report and Road to Health booklet data. While this may not reflect whether a child was HIV-exposed in utero or during the first 18 months of life, it was included as a variable to capture differences in the lived experiences of children and families affected by HIV. Level 2 captures maternal health behaviours prior to and during childbirth, including antenatal care attendance and delivery mode. Attendance at antenatal care is a known predictor of complete and timely childhood vaccination and was measured as the number of antenatal visits reported by the mother.^36 37^ Caesarean section delivery may influence well-care visit attendance, either by increased perceived need for postnatal care or by creating mobility challenges during recovery. In South Africa, caesarean section is not a recommended practice without valid indication.^41^ Caesarean section delivery was identified using records in the Road to Health booklet and supplemented by maternal self-report where missing. Level 3 contains two groups of enabling factors: support-related factors (i.e., access to caregiving support, child and mother cohabitation, access to child support grant and age at which the child’s mother returned to school) and health services factors (i.e., healthcare worker attitudes, waiting time at the clinic and distance to the clinic). Child–mother cohabitation was defined as the child living with their biological mother at least one night per week. Access to caregiving support was defined as mothers receiving help with childcare, household tasks or buying essentials for the child at least once every 2 weeks. Healthcare worker attitudes were captured through reports of whether the mother was shouted at during childbirth. Child cohabitation with their mother and maternal return to school are included as proxy indicators of the mother’s availability to accompany the child to well-care visits. Enabling factors are presented in a cluster at this level, recognising that their interactions may support well-care visit attendance in a complex and interdependent manner. A summary of how explanatory variables were derived from the survey and Road to Health booklets is provided in online supplement 3. The framework addresses factors hypothesised to influence attendance at each individual well-care visit. While prior attendance may affect subsequent attendance and overall retention, this is not represented in the framework to maintain clarity and interpretability.

### Outcome measure

Three indicators of engagement were created: constancy (the proportion of children who attended all eight recommended visits within 4 weeks of the recommended age), few missed visits (the proportion of children with ≤1 missed visits within 4 weeks of the recommended age) and optimal retention (the proportion of children who attended visits within 4 weeks of the recommended age, attended the 18-month visit and missed no more than one visit).^42^

First, we created a variable for attendance for eight well-care visits (i.e., 3–6-day visit, 6, 10 and 14-week visits, and 6, 9, 12 and 18-month visits) aligning with WHO guidance^4^ and the South African schedule^14^ (table 1). The decision to focus on these eight WHO-aligned visits was based on methodological and conceptual considerations. The aim was to assess visit constancy and schedule completion, not visit frequency. Additional visits in the South African schedule between WHO-recommended visits are primarily for growth monitoring and do not introduce new interventions (table 1) and since children are typically caught up on missed care at subsequent visits, any one visit within the interval was considered sufficient to reflect receipt of well-care interventions in line with children’s age. Finally, aligning with the WHO schedule supports comparability with studies beyond South Africa. Attendance was determined by whether a well-care or vaccination date was recorded within specified age thresholds (table 1), ensuring no overlap between consecutive visits while allowing flexibility in scheduling and preventing double-counting.^43 44^ For the first four visits, stricter thresholds aligned with precise age recommendations counted attendance if a visit occurred 1 week before or up to 3 weeks after the recommended age (e.g., 5–8 weeks for the 6-week visit). From 6 months onward, broader thresholds were used to reflect typical scheduling practices, allowing attendance within 1 month after the recommended age (e.g., 25–33 weeks for the 6-month visit).

Second, using these eight well-care visit attendance variables, we created a binary outcome variable for optimal retention in well-care visits up to 18 months. ‘Optimal’ retention (coded as 1) was defined as missing no more than one visit and attending the 18 months. One missed visit prior to the last visit was allowed, as missing at least one visit was common and children caught up on missed services at their next visit.^45^ ‘Suboptimal’ retention (coded as 0) was defined as missing the final visit or missing two or more visits. Optimal retention is the outcome used for examining factors associated with well-care visit attendance, as it is the most relevant indicator of continued contact with well-care services over time and captures both visit constancy and schedule completion.^46^ Additional analyses were conducted using retention up to 12 months as an outcome, defined as missing no more than one visit and attending the 12-month visit within 4 weeks of the recommended age.

### Patient and public involvement

All research tools and data collection methods were developed in consultation with adolescent mothers and an adolescent advisory group.^47^ Researchers and health practitioners in adolescent health in South Africa and sub-Saharan Africa were also consulted.

#### Statistical analysis

Participant characteristics were reported using frequencies and proportions. We explored differences in characteristics by maternal HIV status, used Pearson’s χ² test for categorical variables and the Wilcoxon rank-sum test for continuous variables, with 95% confidence intervals (CI). We calculated the proportion of well-care visits attended and the missed visits. We report retention in well-care visits up to 18 months for three measures of healthcare engagement: visit constancy (i.e., proportion of children who attended all recommended visits within 4 weeks of the recommended age), few missed visits (i.e., proportion of children with any missed visits within 4 weeks of the recommended age vs none) and optimal retention (i.e., proportion of children who completed the schedule within 4 weeks of the recommended age and who missed no more than one visit).

Associations between individual exposure factors and well-care visit retention up to 18 months were examined using univariable logistic regression to estimate odds ratios (OR) and 95% CIs and to obtain p values from the Wald test. Following the hierarchical modelling approach,^48^ we used the conceptual framework (figure 1) to build multivariable logistic regression models. All variables were included in each subsequent model, regardless of their p value in the univariable analysis. In model 1, we examined the association of level 1 variables (socioeconomic and demographic characteristics) with well-care visit retention, treating level 1 variables as potential confounders of the relationship between other level 1 variables and retention. Model 2 added level 2 variables (maternal health-seeking behaviours), adjusting for level 1 factors considered as confounders for the association between maternal health-seeking behaviours and retention. Finally, model 3a and 3b estimated the effects of level 3a (support-related factors) and 3b (health services factors) variables, respectively, adjusting for level 1 and 2 variables, which were retained as confounders in line with the hierarchical modelling approach. Sensitivity analyses were conducted using the same approach to examine associations with retention up to 12 months.

Analyses were conducted using STATA V.17.0. We used stratum-specific estimates and the likelihood-ratio test to assess evidence for effect modification for (1) waiting time at clinic and distance to health facility and (2) food insecurity and access to child support grant. These variables were selected based on theoretical and contextual considerations. The impact of long waiting times on retention may differ according to travel duration to the clinic. Similarly, the likelihood of experiencing food insecurity may be moderated by access to the child support grant. Missing data were coded as an additional category for variables where more than 5% of observations were missing.^49^ This applied to only one variable: number of antenatal care visits attended (14.5% of mothers responded ‘I don’t know’). Further sensitivity analyses were conducted to assess if findings differed for different indicators of well-care visit engagement (i.e., well-care visit constancy and few missed visits).

## Results

### Participant characteristics

A total of 502 children of 481 adolescent mothers were included in this analysis, of whom 51.2% were female (table 2). The median age of mothers at the birth of the oldest living child was 16.9 years (interquartile range (IQR) 15.8, 18.1). Over half (56.8%) of children were born after the introduction of the new vaccination schedule in December 2015. About 28.7% of the children lived in a rural area. Approximately 14.3% of children were reported not to have ever received the child support grant, and 55.2% began receiving the grant at 3 months or older. At the time of pregnancy with their oldest child, 75.6% of mothers had attended or completed at least one secondary school grade (≥9 grade). Most children (93.6%) lived with their mother at least one night a week. Overall, 9.6% of children had experienced at least 1 day in the past week without enough food or milk. All but three children were born in a healthcare facility, with 25.3% born via caesarean section and 12.9% born with low birth weight. Antenatal care coverage was high, with 68.7% of mothers attending five or more appointments. However, initiation was delayed, with fewer than half (44.9%) starting antenatal care in the first trimester. Although postnatal care is delivered during well-care visits, 35.0% of mothers reported not receiving any postnatal care visits after their oldest child’s birth.

**Table 2:** Characteristics of participants

	Total (n=502)	Children of mothers living with HIV* (n=183)	Children of HIV-negative mothers (n=319)	P value†
	Frequency, n (%)			
Socioeconomic and demographic factors				
Sex				
Male	245 (48.8)	89 (48.6)	156 (48.9)	1.0
Female	257 (51.2)	94 (51.4)	163 (51.1)	
Birth cohort				
2009 EPI	217 (43.2)	100 (54.6)	117 (36.7)	<0.001
2015 EPI	285 (56.8)	83 (45.4)	202 (63.3)	
Birth order				
Eldest	472 (94.0)	162 (88.5)	310 (97.2)	<0.001
Second/third oldest	30 (6.0)	21 (11.5)	9 (2.8)	
Residence				
Urban	358 (71.3)	141 (77.0)	217 (68.0)	0.03
Rural	144 (28.7)	42 (23.0)	102 (32.0)	
Child age at grant receipt				
None	71 (14.3)	17 (9.4)	54 (17.2)	0.07
0–4 weeks	88 (17.8)	39 (21.5)	49 (15.6)	
5–14 weeks	63 (12.7)	24 (13.3)	39 (12.4)	
≥3 months	273 (55.2)	101 (55.8)	172 (54.8)	
Maternal age at birth‡ (years, median (IQR))	16.9 (15.8, 18.1)	17.9 (16.4, 18.9)	16.6 (15.7, 17.4)	<0.001§
Maternal education (highest grade attended/completed at age of pregnancy)‡				
Primary (grade ≤8)	112 (24.4)	43 (28.3)	69 (22.5)	0.2
Secondary (grades 9–12)	347 (75.6)	109 (71.7)	238 (77.5)	
Food security				
No	48 (9.6)	30 (16.5)	18 (5.7)	<0.001
Yes	450 (90.4)	152 (83.5)	298 (94.3)	
Cohabitation				
≥1 nights/week	470 (93.6)	173 (94.5)	297 (93.1)	0.5
None	32 (6.4)	10 (5.5)	22 (6.9)	
Birth outcome				
Delivery mode				
Vaginal delivery	375 (74.7)	136 (74.3)	239 (74.9)	0.9
Caesarean section	127 (25.3)	47 (25.7)	80 (25.1)	
Birth weight				
Normal birthweight ≥2500 g	432 (87.1)	157 (86.7)	275 (87.3)	0.9
Low birth weight (<2500 g)	64 (12.9)	24 (13.3)	40 (12.7)	
Maternal health services				
Number of antenatal care visits attended‡				
None	12 (2.5)	5 (3.1)	7 (2.3)	0.01
1–4 appointments	68 (14.4)	15 (9.3)	53 (17.1)	
≥5 appointments	324 (68.6)	108 (66.7)	216 (69.7)	
Don't remember	68 (14.4)	34 (21.0)	34 (11.0)	
Trimester at first antenatal care visit‡				
First	210 (44.9)	75 (47.2)	135 (43.7)	0.2
Second	207 (44.2)	72 (45.3)	135 (43.7)	
Third/during birth	51 (10.9)	12 (7.5)	39 (12.6)	
Number of postnatal care visits attended‡				
None	164 (35.2)	62 (39.5)	102 (33.0)	0.2
≥1 appointment	302 (64.8)	95 (60.5)	207 (67.0)	

* Unconfirmed if these children were HIV-exposed at birth as adolescent mothers may have acquired HIV after child’s birth.

† Obtained using Pearson’s χ² test.

‡ At birth of first order child (n=472).

§ Obtained using Wilcoxon rank-sum test.

EPI, Expanded Programme on Immunisation; IQR, Interquartile range.

HIV prevalence among mothers was 34.3% (n=162). At the time of data collection, 36.5% (n=183) of children had an adolescent mother living with HIV (AMLHIV). AMLHIV were older at the birth of their oldest child than HIV-negative mothers (p<0.001). compared with children of HIV-negative mothers, a higher proportion of children of AMLHIV were born before the schedule change (p<0.001), had siblings (p<0.001), resided in urban areas (p=0.03) and reported food insecurity (p≤0.001). HIV-negative mothers attended more antenatal care visits (p=0.01). There was no other evidence of differences by maternal HIV status in postnatal care attendance, birth outcomes, maternal education or age of child support grant receipt.

#### Well-care visit attendance patterns

**Figure 2:**
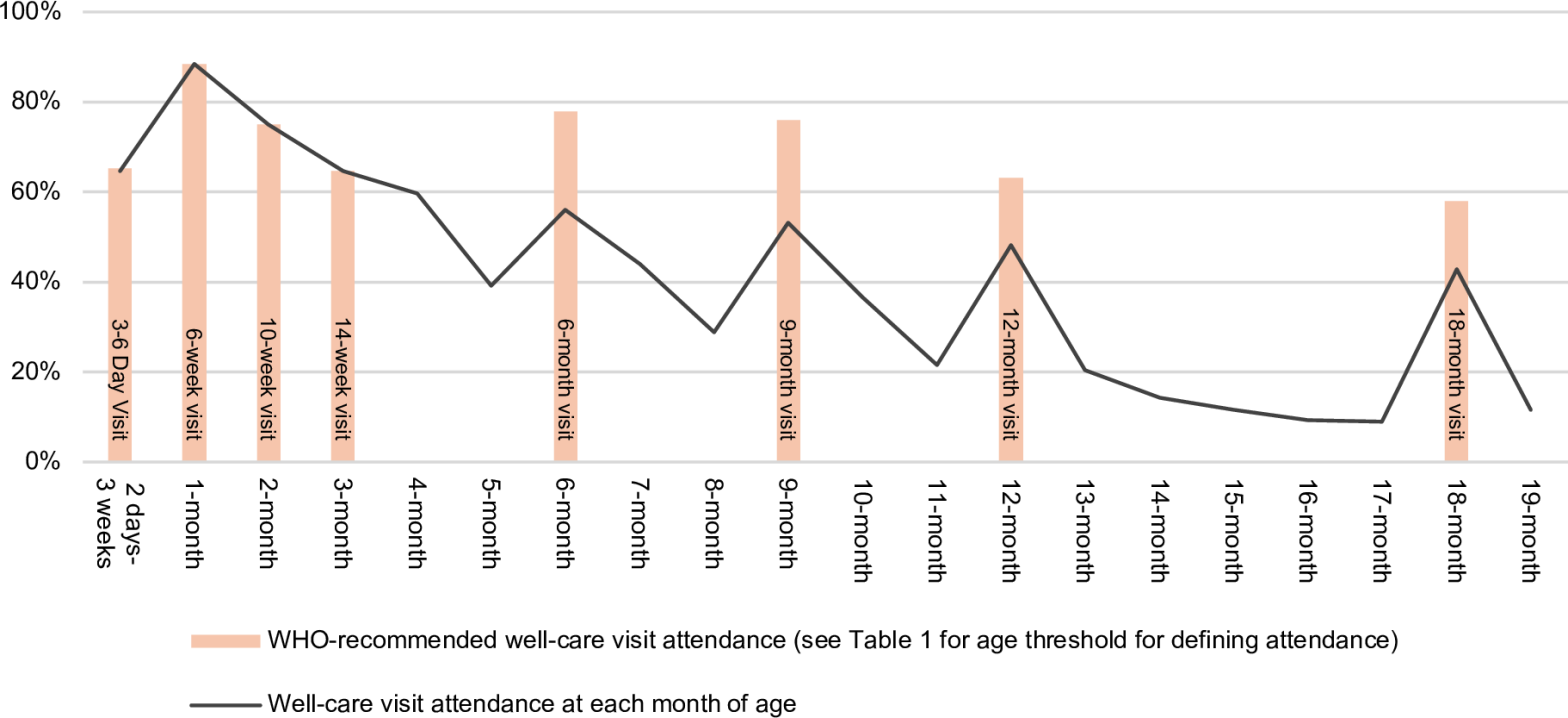
Proportion of children attending well-care visits and/or vaccinations per month of age (line graph) and WHO-recommended well-care visit attendance (see table 1 for age thresholds for attendance) (bar graph).

All but one child in the study had attended at least one well-care visit. This child received only birth vaccinations and had no recorded well-care visits attended in the first 19 months. Figure 2 illustrates well-care visit attendance patterns. The line represents the proportion of children with a record of a well-care visit or vaccination at each month of age. The line graph indicates a general decline in monthly attendance as child age increases, with increases at ages corresponding to the childhood vaccination schedule (6–14 weeks, and 6, 9, 12 and 18 months). The bar graph shows the proportion of well-care visits attended within 4 weeks of the recommended age for eight WHO-recommended well-care visits (3–6-day visit, 6, 10 and 14-week visits and 6, 9, 12 and 18-month visits). As per age thresholds for defining attendance (table 1), 65.1% (95% CI 61.0% to 69.3%) of children had a well-care visit recorded between 2 and 31 days after birth. Attendance at the 6-week visit was the highest with 88.4% (95% CI 85.6% to 91.3%) attending between 5 and 8 weeks old. Attendance declined to 64.7% (95% CI 60.5% to 68.9%) for the 14-week visit before increasing again at the 6- and 9-month visits to 77.9% (95% CI 74.2% to 81.5%) and 75.9% (95% CI 72.1% to 79.7%), respectively. Approximately 63.1% (95% CI 58.9% to 67.4%) of children attended the 12-month visit and 58.0% (95% CI 53.6% to 62.3%) the 18-month visit.

Three indicators of well-care visits engagement up to 18 months were measured (table 3). By 18 months, 19.1% (95% CI 15.7% to 22.6%) of children attended all eight visits within 4 weeks of the recommended age (‘visit constancy’). Approximately 42.2% (95% CI 37.9% to 46.6%) of the children missed no more than one visit by 18 months (‘few missed visits’). Approximately 36.1% (95% CI 31.8% to 40.3%) of children in this study attended the 18-month visit with minimal missed visits (‘retention’).

**Table 3:** Indicators of well-care visit engagement up 18 months (n=502)

Measures of retention	n Proportion (95% CI)
Constancy (0 missed visits)*	96 19.1 (15.7 to 22.6)
Few missed visits (≤1 missed visit)†	212 42.2 (37.9 to 46.6)
Optimal retention (≤1 missed visit and completed schedule)‡	181 36.1 (31.8 to 40.3)

* Proportion of children who attended all recommended visits within 4 weeks of the recommended age.

† Proportion of children with ≤1 missed visits within 4 weeks of the recommended age.

‡ Proportion of children who missed no more than one visit and attended the 18 month visit (i.e., completed the schedule within 4 weeks).

#### Factors associated with retention in the well-care visit schedule

First, we examined the association of well-care visit retention with sociodemographic and economic factors (level 1). In the multivariable analyses, there was evidence that after adjusting for other sociodemographic factors (see model 1 table 4), retention was higher among children living in a rural versus urban area (adjusted odds ratio (aOR)=2.01, 95% CI 1.32 to 3.06) and having a mother with secondary vs primary school education (aOR=2.73, 95% CI 1.60 to 4.65). Additionally, there was weak evidence that retention was associated with food security (aOR=2.18, 95% CI 0.96 to 4.96) and HIV-negative mothers (aOR=0.64, 95% CI 0.40 to 1.02). Next, level 2 (maternal health behaviour) variables were included (see model 2, table 4) and retention was associated with having a caesarean section versus vaginal birth (aOR=1.65, 95% CI 1.05 to 2.60). For level 3a (support-related factors) variables (see model 3 a, table 4), we found weak evidence that retention was associated with receiving the child support grant (aOR=1.71, 95% CI 0.92 to 3.16). Finally, level 3b variables (health service factors) were included (see model 3b, table 4). Retention was associated with longer travel time to the clinic (aOR=0.52, 95% CI 0.28 to 0.96). Similar findings were observed in analyses for retention in the well-care visit schedule up to 12 months (see online supplement 4).

**Table 4:** Frequencies and multivariable analysis for optimal retention in well-care visits up to 18 months

	Total	Optimal retention	OR (95% CI)	P value*	aOR (95% CI)	P value*
	Frequency, n (%)					
	502 (100)	181 (36.1)				
Level 1: Socioeconomic and demographic factors					Model 1†	
Birth cohort						
2009 EPI	217 (43.2)	68 (31.3)	ref		ref	
2015 EPI	285 (56.8)	113 (39.6)	1.44 (0.99 to 2.09)	0.05	1.25 (0.83 to 1.88)	0.3
Residence						
Urban	358 (71.3)	110 (30.7)	ref		ref	
Rural	144 (28.7)	71 (49.3)	2.19 (1.48 to 3.26)	<0.001	2.01 (1.32 to 3.06)	0.001
Maternal age at birth						
≤15	137 (27.3)	50 (36.5)	ref		ref	
16–18	299 (59.6)	109 (36.5)	1.00 (0.66 to 1.52)	0.9	0.75 (0.46 to 1.21)	0.6
19–22	66 (13.1)	22 (33.3)	0.87 (0.47 to 1.62)		0.96 (0.45 to 2.04)	
Maternal education						
Primary	121 (25.1)	10 (8.3)	ref		ref	
Secondary	362 (74.9)	85 (23.5)	2.88 (1.77 to 4.68)	<0.001	2.73 (1.60 to 4.65)	0.001
Maternal HIV status						
Negative	319 (63.5)	133 (41.7)	ref		Ref	
Positive	183 (36.5)	48 (26.2)	0.50 (0.33 to 0.74)	<0.001	0.64 (0.40 to 1.02)	0.08
Food security						
No	48 (9.6)	8 (16.7)	ref		ref	
Yes	450 (89.6)	173 (38.4)	3.12 (1.43 to 6.83)	0.002	2.18 (0.96 to 4.96)	0.06
Level 2: Health behaviour					Model 2‡	
Trimester at first antenatal care visit						
First	227 (45.6)	80 (35.2)	ref		ref	
Second	219 (44)	73 (33.3)	0.92 (0.62 to 1.36)	0.3	1.02 (0.67 to 1.55)	0.2
Third/birth	52 (10.4)	25 (48.1)	1.60 (0.83 to 3.09)		1.81 (0.94 to 3.51)	
Delivery mode						
Vaginal delivery	375 (74.7)	124 (33.1)	ref		ref	
Caesarean section	127 (25.3)	57 (44.9)	1.65 (1.09 to 2.49)	0.02	1.65 (1.05 to 2.60)	0.03
Level 3a: Support-related factors					Model 3a§	
Caregiving support						
No	50 (10.0)	12 (24.0)	ref		ref	
Yes	452 (90.0)	169 (37.4)	1.89 (0.96 to 3.72)	0.05	1.49 (0.66 to 3.35)	0.4
Cohabitation with mother						
≥1 nights/week	32 (6.4)	13 (40.6)	ref		ref	
0 nights/week	470 (93.6)	168 (35.7)	0.81 (0.39 to 1.69)	0.6	0.69 (0.29 to 1.64)	0.4
Access to child support grant						
Never	71 (14.2)	19 (26.8)	ref		ref	
Ever	428 (85.8)	160 (37.4)	1.63 (0.93 to 2.86)	0.08	1.71 (0.92 to 3.16)	0.08
Mother returned to school after pregnancy						
No	143 (28.7)	49 (34.3)	ref		ref	
Yes	341 (68.3)	132 (38.7)	1.21 (0.81 to 1.82)	0.4	0.94 (0.57 to 1.53)	0.8
Level 3b: Health service factors					Model 3b¶	
Distance to clinic (min)						
≤20 min	216 (44.4)	88 (40.7)	ref		ref	
21 to ≤45 min	190 (39.0)	58 (30.5)	0.64 (0.42 to 0.96)	0.1	0.52 (0.33 to 0.83)	0.01
>45 min	81 (16.6)	29 (35.8)	0.81 (0.48 to 1.38)		0.52 (0.28 to 0.96)	
Waiting time at clinic (min)						
<30 min	73 (15.1)	33 (45.2)	ref		ref	
31 to ≤60 min	105 (21.6)	40 (38.1)	0.75 (0.41 to 1.37)	0.3	0.68 (0.34 to 1.35)	0.2
61 min to ≤2 hours	138 (28.5)	49 (35.5)	0.67 (0.37 to 1.19)		0.68 (0.35 to 1.30)	
>2 hours	169 (34.8)	54 (32.0)	0.57 (0.32 to 1.00)		0.60 (0.32 to 1.13)	
Harsh healthcare worker attitudes at birth						
No	390 (80.4)	143 (36.7)	ref		ref	
Yes	104 (21.4)	38 (36.5)	0.99 (0.63 to 1.56)	1.0	0.98 (0.59 to 1.66)	1.0

* Wald test.

† Model 1 adjusted for level 1 (birth cohort, residence, maternal education, maternal HIV status and food security) variables.

‡ Model 2 adjusted for level 1 (birth cohort, residence, maternal education, maternal HIV status and food security) and level 2 (trimester at first antenatal care visit and birth delivery mode) variables.

§ Model 3a adjusted for level 1 (birth cohort, residence, maternal education, maternal HIV status and food security), level 2 (trimester at first antenatal care visit and birth delivery mode) and level 3a (any caregiving support to cohabitation with mother, access to child support grant and mother return to school) variables.

¶ Model 3b adjusted for level 1 (birth cohort, residence, maternal education, maternal HIV status and food security), level 2 (trimester at first antenatal care visit and birth delivery mode) and level 3b (distance to clinic, waiting time at clinic and harsh healthcare worker attitudes at birth) variables.

aOR, Adjusted odds ratio; EPI, Expanded Programme on Immunisation; OR, Odds ratio.

## Discussion

This study examined factors associated with well-care visit retention up to 18 months among children of adolescent mothers in the Eastern Cape, South Africa. Children in the study attended more visits, and at varying times, than recommended by WHO well-care visit guidance.^4^ Attendance peaked at the 6-week visit (88.4%) but declined over time, reaching its lowest at the 18-month visit (58.0%). Only 36.1% of children completed the schedule with no more than one missed visit before 18 months. Retention was associated with rural residence, maternal education, caesarean section and shorter travel time to clinics. We found weak evidence of associations with food security, access to the child support grant and maternal HIV-negative status. These findings should be interpreted with caution, as suboptimal retention in our study was not limited to children with perinatal HIV exposure. Most children experienced suboptimal retention. Targeted strategies for HIV-exposed children may overlook other high-risk groups and the need for universal interventions for all children of adolescent mothers.

While early attendance was relatively high, retention declined steadily from 9 months onward, consistent with other studies of services delivered at well-care visits and PMTCT programmes.^23^,^25^ This limits children’s access to health promotion and prevention services, reducing opportunities for early detection of health issues and timely referrals. As a result, children may miss opportunities for referrals to occupational, speech and physical therapy, which are freely available in South Africa’s public health system. These interventions are particularly important for children of adolescent mothers and those with perinatal HIV exposure, who are at higher risk of cognitive delays.^9 10^

Retention is particularly important for children with perinatal HIV exposure, as the 18-month visit includes confirmatory HIV testing. Lower retention increases the risk of delayed HIV diagnosis and linkage to care. Some studies report lower attendance up to 6 months post partum among children without HIV exposure,^27 34^ while others link positive maternal HIV status to delayed vaccination uptake.^50 51^ Although evidence was weak, findings suggest that children of AMLHIV may have lower retention in well-care visits. Young maternal age is also associated with lower postnatal care attendance,^27^ with barriers including stigma, negative healthcare worker attitudes and lack of transport money.^22^ These challenges may be compounded for AMLHIV, who must also manage their own HIV care and experience poorer health outcomes compared with their nulliparous peers.^52^ Descriptive analyses show that children of AMLHIV were more likely to experience food insecurity (a proxy for household income) and live in urban settings—both factors independently associated with suboptimal retention in regression analysis. Lower retention among children of AMLHIV may be linked to higher financial constraints in households with AMLHIV. An additional financial burden may occur due to more frequent clinic visits for antiretroviral treatment refills, limiting the funds available for transport for their children’s well-care visits.

As reported elsewhere,^27^ low attendance is a concern during the neonatal period. WHO recommends four postnatal contacts for maternal and newborn health: within 24 hours, 48–72 hours, 7–14 days and 6 weeks postpartum.^53^ In this study, only 65.1% of children had at least one contact with primary health facilities between the age of 2–31 days, leaving over a third without contact with health services during this critical risk window. This gap is concerning, given the elevated maternal and neonatal mortality risk among adolescent mothers.^3 6 54^ As postnatal care is also delivered to mothers during well-care visits for children, by proxy this indicates low uptake of postnatal care among adolescent mothers. There remains a critical evidence gap for interventions to promote uptake of postnatal care, particularly for adolescent girls, in Southern Africa.^55^ Future research should include adolescent mothers and their children to better understand and address the barriers they face.

In this study, children born via caesarean section were more likely to attend early well-care visits and remain engaged up to 18 months, suggesting benefits for attendance in the postpartum period and ongoing retention. No association was found between retention and birth weight, suggesting that adverse birth outcomes may not influence retention. Despite reduced mobility following surgery, mothers who deliver through caesarean section may perceive a greater need for postnatal care, increasing attendance to early well-care visits. This supports the possibility that the protective effect of caesarean delivery relates to provider–mother interactions rather than infant health needs. Additionally, increased interaction with healthcare workers during and after a caesarean section may support continued retention beyond the postpartum period. Recent evidence shows that adolescent mothers frequently experience judgement, stigma and limited emotional support during postnatal care, which can undermine engagement.^22^ Mothers who have undergone surgery are likely to receive more attention from healthcare workers by receiving more postnatal guidance and discharge counselling than mothers giving birth by normal vaginal delivery. This increased interaction may have a lasting therapeutic effect, promoting continued attendance to well-care visits.^56^ This association has been overlooked in studies of health-seeking behaviour and warrants further investigation. Future research should explore how provider–mother interactions differ by mode of delivery and examine how the provider–mother interactions experienced by mothers who undergo caesarean sections can be replicated for those who give birth vaginally.

Maternal education is widely recognised as protective for child health, influencing vaccination uptake^57^ and PMTCT retention.^58^ In this study, children of adolescent mothers who reached secondary school were 2.7 times more likely to be retained in well-care visits than those whose mothers had only primary education. Appropriate timing for mothers’ return to school should be considered^59^ as evidence suggests that returning to school can disrupt breastfeeding^60 61^ and mother–child attachment.^62^ However, we found no evidence that mothers returning to school after pregnancy affected children’s retention. Children of mothers who returned to school in this study may be able to maintain access to well-care visits due to availability of caregiving support which facilitates their children’s access to health services.^63^ Kinship networks are central to sustaining postnatal care engagement among adolescent mothers.^22^ Although informal childcare is common in South Africa,^64^ formal childcare services remain crucial for supporting young mothers and improving outcomes for their children.^65^

Structural inequalities in South Africa drive disparities in healthcare access, particularly among black rural populations, where adolescent childbearing is most concentrated and affects those in the lowest wealth quintile.^66^ Though evidence was weak, we found that food security and access to the child support grant were associated with better retention, aligning with research showing that wealth is a key determinant of healthcare access.^67^ To expand access, South Africa is re-engineering primary healthcare and integrating community health workers (CHWs) into the health system.^68^ This involves CHWs in delivering maternal and child health services, particularly for underserved rural areas. In this study, children in rural areas were more likely to have optimal retention compared with those living in urban areas. Despite poorer availability and accessibility of health services in rural areas, the quality and acceptability of care may be higher than in urban settings.^69 70^ Lower patient loads in rural clinics may contribute to perceived efficiency, whereas urban clinics have higher patient volumes resulting in longer waiting times.^70^ Our finding highlighting improved retention in rural areas may indicate more effective rural-friendly models, such as mobile clinics and CHW outreach. Addressing the burden of high patient loads in urban clinics is critical for improving service delivery. Additionally, leveraging investments in CHWs to reach children of adolescent mothers in urban settings is essential. Further research should explore why retention is better in rural areas and how these insights can inform strategies to promote retention in urban areas.

Although improving service quality in urban areas may enhance retention, indicators of poor service quality were not associated with retention in this study. One in five mothers reported being shouted at during childbirth, and 63.3% waited over an hour for well-care visits—both known barriers to adolescent access to health services.^17 18^ Mothers’ commitment to prioritising well-care visit attendance may supersede these barriers.^26 31^ Nonetheless, as child age increases, competing demands may reduce attendance and contribute to poor retention. Lack of about well-care services, vaccine stock-outs or dissatisfaction with care could also contribute to poor retention. Reinforcing the importance of well-care visits and improving service quality may support retention. Our findings support calls to enhance the quality and delivery of well-care services.^71 72^

### Strengths and limitations

A key strength of this study is its underpinning in qualitative interviews with adolescent mothers, which informed the development of a conceptual framework to assess well-care visit retention. Data on sociodemographic, maternal health behaviours, support-related and healthcare service-related factors allowed for a comprehensive analysis while adjusting for known determinants (e.g., maternal education). This approach ensured a clearer interpretation of retention factors by accounting for distal factors that might otherwise be underestimated due to more proximate factors.^48^ Combining well-care visit and vaccination dates improved the accuracy of measuring attendance at primary healthcare facilities. This approach addresses potential missing data in well-care visit records and prevents misclassification of missed visits due to vaccine stock-outs by distinguishing supply-related missed vaccinations from actual missed visits (online supplement 5). This study focused on eight well-care visits aligned with the WHO schedule, capturing both attendance constancy and schedule completion. Although children attended more visits than WHO recommends, attendance spikes at key intervals validated our approach of focusing on eight WHO-aligned visits. Finally, using health facility and community-based recruitment reduced selection bias and improved the generalisability of findings, particularly for children of adolescent mothers in periurban and rural areas, who may not have regular access to health services. Findings are generalisable to children of adolescent mothers (10–19 years old) living in periurban and rural areas in the Eastern Cape, including those affected by HIV. While generalisability to other provinces may be limited due to differences in health service infrastructure and norms, these findings offer valuable insights for similar contexts across South Africa where adolescent mothers face comparable barriers to access.

While the temporal direction of exposures measured before childbirth (such as maternal health-seeking behaviour and education) provides evidence for potential causal pathways to retention, other risk factors were measured cross-sectionally, limiting our ability to explore causal links between support-related and healthcare service factors and the outcome. Unmeasured factors, such as maternal knowledge about well-care visits and vaccine stock-outs, could further explain variations in retention. Potential response bias may have led to an underestimation of the impact of service quality on retention, as mothers who experienced harsh treatment from healthcare workers during childbirth may have been less willing to disclose it. Maternal return to school was measured only at baseline, which may have led to non-differential misclassification and underestimation of the number of mothers who returned to school during the 18-month observation period. However, exploratory analyses among children over 19 months old at baseline did not show an association between maternal return to school and well-care visit retention, suggesting minimal impact on the findings. Similarly, potential non-differential misclassification of residence, measured cross-sectionally and possibly affected by population mobility, is unlikely to have meaningfully influenced the results. Lastly, a small proportion of eligible children was excluded due to missing or damaged Road to Health booklets. Compared with those included, excluded children did not differ on factors associated with well-care retention in this study (see online supplement 6). However, children excluded from the analysis were slightly older at the time of data collection, likely because older children are more likely to have lost or damaged booklets, either due to prolonged use or reduced need for the booklet when they are older. In addition, excluded children were also more likely to be born with low birth weight; however, we found no association between birth weight and retention.

## Conclusions

This study builds on PMTCT retention research by extending the focus to well-care services and including high-risk children of adolescent mothers without perinatal HIV exposure. Our findings highlight the need for universal strategies to improve retention in child health services. This aligns with efforts to integrate PMTCT and HIV services with well-care services in primary care. High-quality, accessible and reliable well-care services will be crucial in improving retention and health outcomes for children and their adolescent mothers.

## Supplementary material

10.1136/bmjopen-2025-106412 online supplemental file 1

## Data Availability

Data are available on reasonable request.
