# Supplementary material for: Factors Associated with Retention in Routine Well-Care Visits Among Children of Adolescent Mothers Living With and Without HIV: A Community-Based Study in the Eastern Cape, South Africa
Source: BMJ Open. 2025 Dec 31;15(12):e106412. doi: 10.1136/bmjopen-2025-106412 (PMC12766768; doi:10.1136/bmjopen-2025-106412)
Supplement: online supplemental file 1 [file bmjopen-15-12-s001.pdf]

SUPPLEMENTAL MATERIALS

Supplement 1: Research partnership reflexivity statement

**Research partnership:** This publication is one of three pieces of original research undertaken as part of the lead author's doctoral project at the London School of Hygiene & Tropical Medicine (LSHTM), which is embedded within HEY BABY (Helping Empower Youth Brought up in Adversity with their Babies and Young Children) — a cohort study (Principal Investigators: Prof Lucie Cluver and A/Prof Elona Toska) conducted by the Accelerate Research Hub (The Accelerate Hub). The Accelerate Hub is a research partnership co-led by the University of Cape Town (UCT) and University of Oxford (Oxford), with collaborative involvement from LSHTM. The Accelerate Hub generates policy-relevant evidence to improve outcomes for children, adolescents, and young people across Eastern and Southern Africa. The doctoral researcher has a formal affiliation with Oxford and UCT, and this work reflects an interdisciplinary collaboration across the three partner institutions. Visit the research collaboration website for more information: https://www.acceleratehub.org/.	
**Study conceptualisation**	
1. How does this study address local research and policy priorities?<br>2. How were local researchers involved in study design?	Prior to conceptualising the HEY BABY study, the research team conducted a large-scale community-traced adolescent antiretroviral adherence study called Mzantsi Wakho. Research objectives and methods for the HEY BABY study stemmed from evidence generated through Mzantsi Wakho and ongoing collaboration with stakeholders.<br><br>The HEY BABY study was designed and conceptualised in collaboration with South African Departments of Health, Basic Education, and Social Development, UNICEF and UNFPA, the World Health Organization, REPSSI, Paediatric AIDS Treatment for Africa, and community-based organisations: the Keiskamma Trust, Kheth'Impilo, Beyond Zero, the Relevance Network and Small Projects Foundation.<br><br>For more information about the HEY BABY study please visit the study website: https://www.heybaby.org.za/
**Research management**	
3. How has funding been used to support the local research team?	UCT and Oxford are co-recipients of funding awarded for the HEY BABY study. This funding supports a fieldwork team based in the Eastern Cape, South Africa, as well as an international team of researchers, including early-career academics, a policy engagement and advocacy team, and operations staff.<br><br>A central activity of the Accelerate Hub is to strengthen research capacity among early-career academics, supporting locally led research and evidence-informed policy in Africa. Early-career academics are mentored and receive training in areas ranging from statistical methods to policy engagement. This builds research skills, fosters academic collaboration, and ensures African scholars are leading knowledge generation for the region.
**Data acquisition and analysis**	
4. How are research staff who conducted data collection acknowledged?<br>5. Do all members of the research partnership have access to study data?<br>6. How was data used to develop analytical	Researchers and fieldwork staff who contributed to data collection are acknowledged in all research outputs. Those who meet the International Committee of Medical Journal Editors Recommendations (ICMJE) criteria for authorship, including Co-Investigators and individuals who substantively contributed to the conceptualisation of the study, research tools, data collection and data cleaning, are invited to co-author research outputs. All researchers affiliated with the HEY BABY study and the Accelerate Hub have access to study data and are actively encouraged to use it for analysis and publication. Equitable use of data and transparency in authorship is a core principle and value of the Accelerate Hub. Data users have opportunity to receive mentorship and

skills within the partnership?	support to develop analytical skills, including through structured internal training sessions, collaborative analysis, writing retreats and access to external learning opportunities.
**Data interpretation**	
7. How have research partners collaborated in interpreting study data?	During the final stages of analysis, feedback was sought from South Africa-based researchers at a departmental seminar at UCT. Additionally, the lead author presented the research-in-progress to the fieldwork team to draw on their expertise, ensuring that interpretation of the data was informed by contextual knowledge from those with direct experience with adolescent mothers in the Eastern Cape.
**Drafting and revising for intellectual content**	
8. How were research partners supported to develop writing skills?<br>9. How will research products be shared to address local needs?	In line with other capacity building efforts, the Accelerate Hub actively supports early-career researchers and partners to develop writing skills through involvement in a range of research outputs, including policy briefs, commentaries, and peer-reviewed publications.<br><br>Findings are integrated into ongoing policy engagement and advocacy activities and disseminated through the Hub's established policy partnerships. For example, study results will be presented at an upcoming seminar hosted by a local NGO focused on strengthening Early Childhood Development services in South Africa.
**Authorship**	
10. How is the leadership, contribution and ownership of this work by LMIC researchers recognised within the authorship?<br>11. How have early career researchers across the partnership been included within the authorship team?<br>12. How has gender balance been addressed within the authorship?	This paper is led by a UK-based early-career researcher and doctoral candidate at LSHTM, who holds a formal affiliation at UCT. All co-authors are senior academic mentors based in the UK or South Africa, including one co-author based at UCT. Contributions to study conceptualisation, data analysis, and interpretation were led by the first author with mentorship support from co-authors.<br><br>In line with ICJME criteria, all co-investigators and early-career researchers who contributed substantively were invited to co-author; others are acknowledged for their contributions.<br><br>Given the study's focus on maternal and child health and adolescent motherhood, the all-female authoring team (including women who are also mothers) offers gender-aligned perspectives to the research topic.
**Training**	
13. How has the project contributed to training of LMIC researchers?	As described capacity building is a core activity and objectives of the Accelerate Hub.
**Infrastructure**	
14. How has the project contributed to improvements in local infrastructure?<br>15. What safeguarding procedures were used to protect local study participants and researchers?	The research partnership does not aim to improve local infrastructure. The team are committed to ensuring ethical and safeguarding practices for participants and researchers. The HEY BABY study adheres to a participant referral protocol which is used to facilitate referrals to health and social services (available at www.heybaby.org.za/research). More information on safeguarding policies is available on the Accelerate Hub website. https://www.acceleratehub.org/.<br><br>Data protection mechanisms are in place to ensure compliance with South Africa's Protection of Personal Information Act (POPIA) and the EU's General Data Protection Regulation (GDPR), protecting the rights and personal data of children and adolescents.

Supplement 2: Sampling flowchart

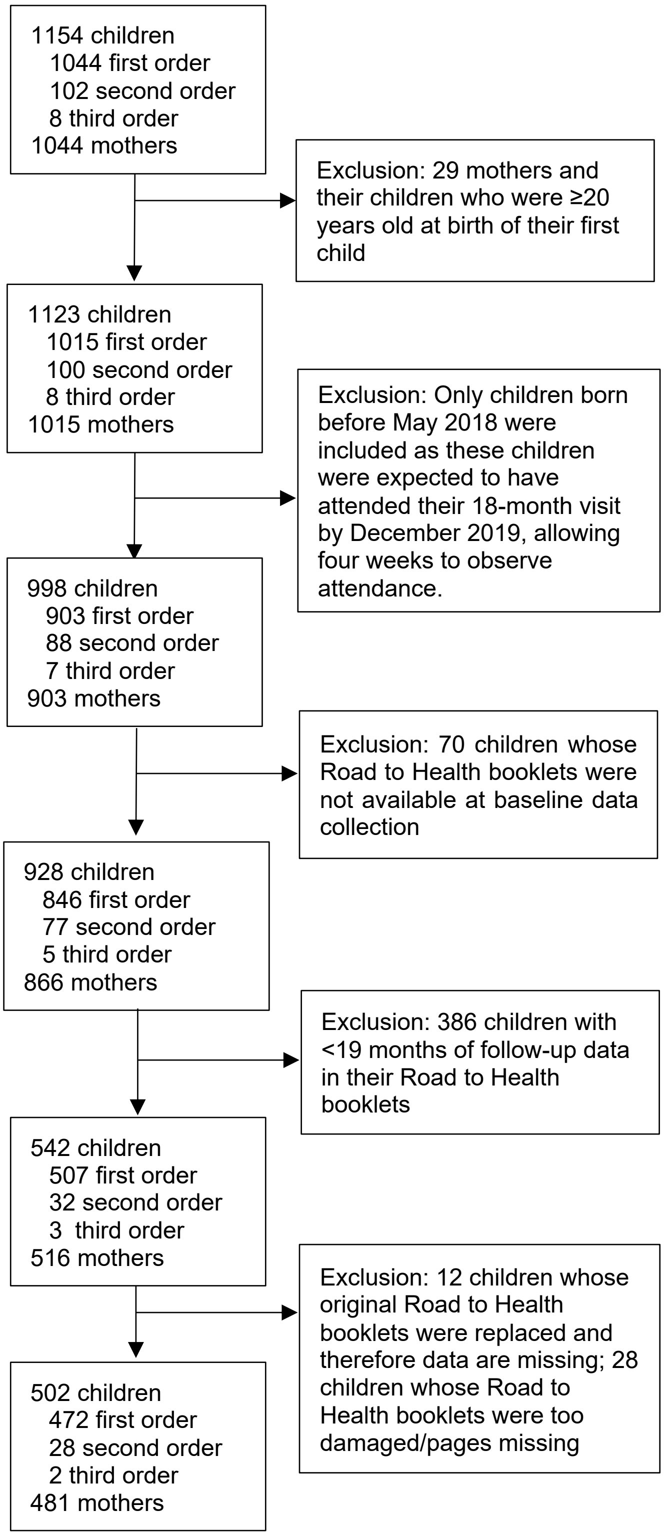

Supplement 3: Summary of variables and coding

Variable	Survey question	Coding	Source
Child Sex	Is [child] a girl or a boy?	1=Male<br>2=Female	Adolescent Parent Questionnaire*
Birth cohort	A binary variable created using the child's date of birth to categorise children by whether they were before and after the Expanded Programme for Immunization (EPI) schedule was updated to include two additional vaccinations (at 6 and 12 months). Children born on or after 1 December 2015 we categorised as being born after the schedule change.	1=2009 EPI<br>2=2015 EPI	Road to Health booklet*
Sibling	Derived from survey response from Adolescent Parent Questionnaire and verified against information children's road to health cards about their birth order.	0=Only-child<br>1=Sibling	Child health record
Birth order	Children in the study were categorised by birth order, with the oldest living child at the time of recruitment classified as the first-order child. Derived from survey response from Adolescent Parent Questionnaire and verified against information children's road to health cards about their birth order.	1=Only-child<br>1=Sibling	Road to Health booklet
Residence	Research assistant recorded if participant lives in either a rural or urban area based on participant's address at data collection.	0=Urban<br>1= Rural	Adolescent Parent Questionnaire
Child age at child support grant receipt	How old was [child] when you started getting the child support grant? Recorded in years, months and weeks OR 'I am not receiving the child support grant for them'	0=None<br>1=0-4 weeks old<br>2= 5-14 weeks old<br>3=≥3 months old	Adolescent Parent Questionnaire
Maternal age at birth	Computed by subtracting mother's date of birth from child's date of birth using the *datediff* command with "month" option.	1=≤15 years<br>2=16-18 years<br>3=19-22 years	Road to Health booklet*
Maternal education (highest grade reached at time of pregnancy)	Measure of the highest school grade mothers reached at the time of pregnancy.<br>What is the highest grade you have passed?<br>What grade were you in when you got pregnant with [child]?	1=Primary (Grade ≤8)<br>2=Secondary (Grade 9-12)<br>0=Unknown	Adolescent Parent Questionnaire
Feeding in first 6 months of life	How was child fed in the first six months of their life?<br>☐ Breastfeeding and baby formula<br>☐ Baby formula only<br>☐ Breastfeeding only<br>☐ Other milk or water	1==Breastfeeding and baby formula<br>2==Baby formula only<br>3==Breastfeeding only<br>4==Other milk or water	Adolescent Parent Questionnaire
Exclusive breastfeeding in first 6 months of life	Derived based on responses from following three question and child's age at a time of survey:<br><br>How was child fed in the first six months of their life?<br>☐ Breastfeeding and baby formula<br>☐ Baby formula only<br>☐ Breastfeeding only<br>☐ Other milk or water<br><br>How long was child breastfed?<br>☐ Still being breastfed<br>☐ Breastfeeding has gradually stopped<br>☐ BF stopped completely<br>☐ Child was never breastfed<br><br>How old was child when they stopped being breastfeed?<br>☐ This child was never breastfed<br>☐ Less than 6 months<br>☐ 6-12 months<br>☐ 1 year or older<br>☐ Still being breastfed	0== Never Exclusive breastfed<br>1== Exclusive Breastfeeding for 6 months<br>2== Exclusive Breastfeeding cessation <6 months	Adolescent Parent Questionnaire
Maternal HIV status	Variables created using self-report and verified against child health records.	0=HIV-Negative<br>1=Mother living with HIV	Road to Health booklet*

Food security (child only)	How many days in the past week, was there not enough food or milk for [child] at home? [Number of days 0-7]<br><br>Food insecurity was defined as experiencing at least one day without enough food at home in the past week.	0=Food insecure<br>1=Food secure	Adolescent Parent Questionnaire
Cohabitation with mother	Does this child live with you?<br>☐ Yes, all the time<br>☐ Yes, 4 or more nights a week<br>☐ Yes, but less than 4 nights a week<br>☐ No	0=0 nights/week<br>1=≥1 nights/week	Adolescent Parent Questionnaire
Delivery mode	Extracted from relevant field in Road to Health booklet and verified with survey questionnaire where missing in Road to Health booklet:<br><br>How did you give birth to your baby?<br>☐ At home<br>☐ In the hospital/clinic (natural birth)<br>☐ In the hospital/clinic (c-section)	1=Vaginal delivery<br>2=Caesarean section	Road to Health booklet*
Birthweight	Extracted from relevant field in Road to Health booklet.	0=Normal birthweight ≥2500g<br>1=Low birthweight <2500g	Road to Health booklet*
Trimester at first antenatal care visit	When was the first time you had clinic/hospital appointment when pregnant with [child]?<br>☐ First trimester (1-3 months pregnant)<br>☐ Second trimester (4-6 months pregnant)<br>☐ Third trimester (7-9 months pregnant)<br>☐ I don't remember but while I was pregnant<br>☐ I did not go to the clinic during pregnancy – just during or after birth	1=First trimester<br>2=Second trimester<br>3=Third trimester<br>4=During birth	Adolescent Parent Questionnaire
Number of antenatal care visits attended	When you were pregnant with [child], how many pregnancy-related/ antenatal appointments at the clinic or hospital did you attend?<br>☐ 9 or more<br>☐ 5 to 8 appointments<br>☐ 2 to 4 appointments<br>☐ 1 appointment<br>☐ None<br>☐ I don't remember how many appointments	0=None<br>1=1-4 appointments<br>2=≥5 appointments<br>3=Don't remember	Adolescent Parent Questionnaire
Number of postnatal care visits attended	After you were discharged, how many times did you have a health-check about your health as a young mother at a hospital or clinic?<br>☐ Once<br>☐ Twice<br>☐ More than 2 times<br>☐ None	0=None<br>1=≥1 appointment	Adolescent Parent Questionnaire
Any caregiving support	Composite variable if response was 'yes' to any of the following questions:<br><br>Does anyone help you look after [child] at home (without you) at least once every 2 weeks?<br>☐ Yes<br>☐ No<br><br>Does anyone help you buy things for [child], like nappies, food, and clothes at least once every 2 weeks?<br>☐ Yes<br>☐ No<br><br>Does anyone help you with washing or preparing food for [child] at least once every 2 weeks?<br>☐ Yes<br>☐ No	0=None<br>1=Yes	Adolescent Parent Questionnaire
Mother's return to school	How old was [child] when you went back to school? Recorded in years and months OR 'I did not go back to school'.	0=No<br>1=Yes	Adolescent Parent Questionnaire

Access to child support grant	How old was [child] when you started getting the child support grant? Recorded in years, months and weeks OR ‘I am not receiving the child support grant for them’<br><br>Variable used to assess if child ever received the grant based on response.	0=Never<br>1=Ever<br>2=Unknown	Adolescent Parent Questionnaire
Distance to clinic (mins)	How long does it usually take to get to the clinic? Recorded in hours and minutes.	1=≤20 mins<br>2=21 - ≤45 mins<br>3= >45mins<br>4=Unknown	Adolescent Parent Questionnaire
Waiting time at clinic (mins)	In the last year, how long did you usually wait to see the nurse/ doctor at the clinic? Recorded in hours and minutes.	1=<30 mins<br>2=31-≤60 mins<br>3=61 mins - ≤2 hours<br>4=>2hrs<br>5=Don’t remember	Adolescent Parent Questionnaire
Shouted at by healthcare worker at Birth	Variable derived from response to question below.<br><br>Please tell us how the healthcare providers made you feel during the birth of [child]?<br>I was shouted at birth<br>☐ Not at all<br>☐ Sometimes<br>☐ Most of the time	0=No (Not at all)<br>1=Yes (sometimes/most of the time)	Adolescent Parent Questionnaire

*Verified using other sources of data including the Adolescent Parent Questionnaire or Road to Health booklet data.

Supplement 4: Factors associated with retention in the well-care visit schedule up to 12 months

	Total	Optimal retention	OR (95% CI)	p-value*	aOR (95% CI)	p-value*
	Frequency, n (%)					
	502 (100)	222 (44.2)				
**Level 1: Socio-economic & demographic factors**					**Model 1[1]**	
**Birth cohort**						
2009 EPI	217 (43.2)	68 (31.3)	ref		ref	
2015 EPI	285 (56.8)	113 (39.6)	1.64 (1.15, 2.36)	0.01	1.4 (0.95, 2.08)	0.09
**Residence**						
Urban	358 (71.3)	110 (30.7)	ref		ref	
Rural	144 (28.7)	71 (49.3)	1.69 (1.14, 2.49)	0.01	1.64 (1.09, 2.48)	0.02
**Maternal age at birth**						
≤15	137 (27.3)	50 (36.5)	ref		ref	
16-18	299 (59.6)	109 (36.5)	1.12 (0.75, 1.69)	0.9	0.87 (0.54, 1.38)	1.0
19-22	66 (13.1)	22 (33.3)	1.07 (0.59, 1.93)		1.11 (0.54, 2.28)	
**Maternal education**						
Primary	121 (25.1)	37 (30.6)	ref	<0.001	ref	0.001
Secondary	362 (74.9)	183 (50.6)	2.32 (1.50, 3.60)		2.29 (1.42, 3.72)	
**Maternal HIV status**						
Negative	319 (63.5)	133 (41.7)	ref		ref	
Positive	183 (36.5)	48 (26.2)	0.63 (0.44, 0.92)	0.02	0.82 (0.53, 1.26)	0.4
**Food Security**						
No	48 (9.6)	8 (16.7)	ref		ref	
Yes	450 (89.6)	173 (38.4)	2.29 (1.18, 4.45)	0.01	1.72 (0.85, 3.46)	0.1
**Level 2: Health behaviour**					**Model 2[2]**	
**Trimester at first antenatal care visit**						
First	227 (45.6)	96 (42.3)	ref		ref	
Second	219 (44.0)	95 (43.4)	1.05 (0.72, 1.52)	0.2	1.17 (0.79, 1.75)	0.1
Third	52 (10.4)	28 (53.8)	1.59 (0.87, 2.92)		1.76 (0.91, 3.37)	
**Delivery mode**						
Vaginal delivery	375 (74.7)	160 (42.7)	ref		ref	
Caesarean section	127 (25.3)	62 (48.8)	1.28 (0.86, 1.92)	0.23	1.21 (0.78, 1.88)	0.4
**Level 3a: Support-related factors**					**Model 3a[3]**	
**Caregiving support**						
No	50 (10.0)	12 (24.0)	ref		ref	
Yes	452 (90.0)	169 (37.4)	1.33 (0.73, 2.42)	0.4	1.19 (0.57, 2.48)	0.7
**Cohabitation with mother**						
≥1 nights/week	32 (6.4)	13 (40.6)	ref		ref	
0 nights/week	470 (93.6)	168 (35.7)	1.17 (0.56, 2.43)	0.7	0.98 (0.43, 2.26)	1.0
**Access to child support grant**						
Never	71 (14.2)	19 (26.8)	ref		ref	
Ever	428 (85.8)	160 (37.4)	1.65 (0.98, 2.80)	0.06	1.95 (1.09, 3.48)	0.02
**Mother returned to school**						
No	143 (28.7)	49 (34.3)	ref		ref	
Yes	341 (68.3)	132 (38.7)	1.22 (0.82, 1.81)	0.3	1.12 (0.7, 1.78)	0.6
**Level 3b: Health service factors**					**Model 3b[4]**	
**Distance to clinic (mins)**						
≤20 mins	216 (44.4)	88 (40.7)	ref		ref	
21 - ≤45 mins	190 (39.0)	58 (30.5)	0.80 (0.54, 1.18)	1.4	0.75 (0.48, 1.15)	0.09
>45mins	81 (16.6)	29 (35.8)	0.85 (0.51, 1.42)		0.64 (0.36, 1.16)	
**Waiting time at clinic (mins)**						
<30 mins	73 (15.1)	33 (45.2)	ref		ref	
31-≤60 mins	105 (21.6)	40 (38.1)	0.76 (0.42, 1.39)	0.2	0.69 (0.36, 1.33)	0.2
61 mins-≤2hrs	138 (28.5)	49 (35.5)	0.69 (0.39, 1.22)		0.72 (0.38, 1.35)	
>2hrs	169 (34.8)	54 (32.0)	0.56 (0.32, 0.97)		0.61 (0.33, 1.11)	
**Harsh healthcare worker attitudes at birth**						
No	390 (80.4)	143 (36.7)	ref		ref	
Yes	104 (21.4)	38 (36.5)	0.80 (0.51, 1.24)	0.3	0.79 (0.48, 1.29)	0.4

[1]Model 1 adjusted for level 1 (birth cohort, residence, maternal education, maternal HIV status and food security) variables.
[2]Model 2 adjusted for level 1 (birth cohort, residence, maternal education, maternal HIV status and food security) and level 2 (trimester at first antenatal care visit and birth delivery mode) variables.
[3]Model 3a adjusted for level 1 (birth cohort, residence, maternal education, maternal HIV status and food security), level 2 (trimester at first antenatal care visit and birth delivery mode), and level 3a (any caregiving support, cohabitation with mother, access to child support grant and mother return to school) variables.
[4]Model 3b adjusted for level 1 (birth cohort, residence, maternal education, maternal HIV status and food security), level 2 (trimester at first antenatal care visit and birth delivery mode), and level 3b (distance to clinic, waiting time at clinic and harsh healthcare worker attitudes at birth) variables.
*wald-test

Supplement 5: Comparison of well-care attendance estimates using vaccination dates only vs vaccination and well-care visit dates (n=502)

Well-care visit schedule	Age threshold for attendance	Well-Care Visit Attendance: Vaccination and well-care visit dates (a)	Vaccination dates only (b)	Difference between estimates (a-b)[1]
		n (%)	n (%)	n (%)
3-6 Days	2 to 31 days	327 (65.1)	139 (27.7)[2]	188 (37.5)
6-week	5 to 8 weeks	444 (88.4)	439 (87.5)	5 (1.0)
10-week	9 to 12 weeks	376 (74.9)	360 (71.7)	16 (3.2)
14-week	13 to 16 weeks	325 (64.7)	313 (62.4)	12 (2.4)
6-month	25 to 33 weeks	391 (77.9)	254 (50.6)	137 (27.3)[3]
9-month	38 to 47 weeks	381 (75.9)	342 (68.1)	39 (7.8)
12-month	51 to 59 weeks	317 (63.1)	201 (40.0)	116 (23.1)[3]
18-month	77 to 86 weeks	291 (58.0)	246 (49.0)	45 (9.0)

1: Calculation of instances where a well-care visit was attended, but no vaccines were administered.
2: Vaccines given at 0–1 days of age are not included in the numerator.
3: The higher discrepancy is likely due to 43.2% of children in the cohort being born during a period when the vaccination schedule did not recommend vaccines at 6 and 12 months.

Supplement 6: Comparison of characteristics of participants with and without Road to Health booklets

	Total (n=542)	Road to Health booklet available (n=502)	Road to Health booklet not available (n=40)	p-value[2]
		Frequency, n (%)		
**Socio-economic & demographic factors**				
**Sex**				
Male	260 (48)	245 (48.8)	15 (37.5)	0.2
Female	282 (52)	257 (51.2)	25 (62.5)	
**Child age at data collection** (years, median [IQR])	2.4 [1.7, 3.3]	2.3 [1.7, 3.2]	3.1 [2.3, 3.7]	0.01
**Birth cohort**				
2009 EPI	245 (45.2)	217 (43.2)	28 (70)	0.001
2015 EPI	297 (54.8)	285 (56.8)	12 (30)	
**Birth order**				
Eldest	507 (93.5)	472 (94)	35 (87.5)	0.1
Second/Third oldest	35 (6.5)	30 (6)	5 (12.5)	
**Residence**				
Urban	384 (70.8)	358 (71.3)	26 (65)	0.4
Rural	158 (29.2)	144 (28.7)	14 (35)	
**Child age at grant receipt**				
None	79 (14.8)	71 (14.3)	8 (20)	0.2
0-4 weeks	99 (18.5)	88 (17.8)	11 (27.5)	
5-14 weeks	69 (12.9)	63 (12.7)	6 (15)	
≥3 months	288 (53.8)	273 (55.2)	15 (37.5)	
**Maternal age at birth[3]** (years, median [IQR])	16.9 [15.8, 18.1]	16.9 [15.8, 18.1]	17.2 [15.8, 18.5]	0.7[4]
**Maternal education (highest grade attended/completed at age of pregnancy)[3]**				
Primary (Grade ≤8)	125 (25.4)	112 (24.4)	13 (38.2)	0.07
Secondary (Grade 9-12)	368 (74.6)	347 (75.6)	21 (61.8)	
**Food Security**				
No	486 (90.3)	48 (9.6)	4 (10)	0.9
Yes	52 (9.7)	450 (90.4)	36 (90)	
**Maternal HIV status[5]**				
Negative	339 (62.5)	319 (63.5)	20 (50)	0.09
Positive	203 (37.5)	183 (36.5)	20 (50)	
**Cohabitation**				
≥1 nights/week	507 (93.5)	470 (93.6)	37 (92.5)	0.8
None	35 (6.5)	32 (6.4)	3 (7.5)	
**Birth outcome**				
**Delivery mode**				
Vaginal delivery	406 (74.9)	375 (74.7)	31 (77.5)	0.7
Caesarean section	136 (25.1)	127 (25.3)	9 (22.5)	
**Birth weight**				
Normal birthweight ≥2500g	456 (85.7)	432 (87.1)	24 (66.7)	0.001
Low birthweight (<2500g)	76 (14.3)	64 (12.9)	12 (33.3)	
**Maternal health services**				
**Number of antenatal care visits attended[3]**				
None	14 (2.8)	12 (2.5)	2 (5.7)	0.5
1-4 appointments	72 (14.2)	68 (14.4)	4 (11.4)	
≥5 appointments	346 (68.2)	324 (68.6)	22 (62.9)	
Don't remember	75 (14.8)	68 (14.4)	7 (20)	
**Trimester at first antenatal care visit[3]**				
First	229 (45.5)	210 (44.9)	19 (54.3)	0.6
Second	220 (43.7)	207 (44.2)	13 (37.1)	
Third/During birth	54 (10.7)	51 (10.9)	3 (8.6)	
**Number of postnatal care visits attended[3]**				
None	173 (34.6)	164 (35.2)	9 (26.5)	0.3
≥1 appointment	327 (65.4)	302 (64.8)	25 (73.5)	

[1]Unconfirmed if these children were HIV-exposed at birth as adolescent mothers may have acquired HIV after child's birth; [2]Obtained using Pearson's chi-squared test; [3]At birth of first order child (n=507); [4]Obtained using Wilcoxon rank-sum test; [5]Unconfirmed if these children were HIV-exposed at birth as adolescent mothers may have acquired HIV after child's birth.
