# Supplementary material for: Factors Associated with Retention in Routine Well-Care Visits Among Children of Adolescent Mothers Living With and Without HIV: A Community-Based Study in the Eastern Cape, South Africa
Source: BMJ Open. 2025 Dec 31;15(12):e106412. doi: 10.1136/bmjopen-2025-106412 (PMC12766768; doi:10.1136/bmjopen-2025-106412)
Supplement: Supplementary file 2 [file bmjopen-2025-106412.reviewer_comments.pdf]

PEER REVIEW HISTORY

BMJ Open publishes all reviews undertaken for accepted manuscripts. Reviewers are asked to complete a checklist review form and are provided with free text boxes to elaborate on their assessment. These free text comments are reproduced below.

ARTICLE DETAILS

Title (Provisional)

Factors Associated with Retention in Routine Well-Care Visits among Children of Adolescent Mothers living with and without HIV: A community-based study in the Eastern Cape, South Africa

Authors

Wittesaele, Camille; Toska, Elona; Cluver, Lucie Dale; Weiss, Helen Anne; Doyle, Aoife Margaret

---

VERSION 1 - REVIEW

**Reviewer**	**1**
**Name**	**Kabiru, Caroline**
**Affiliation**	**African Population and Health Research Center**
**Date**	**04-Jul-2025**
**COI**	**I have partnered with one of the authors on a proposal in the recent past**

In this manuscript, the authors examine the factors that are associated with optimal well-care child visit retention for children of adolescent mothers. The paper is well written and offers valuable insights to inform future research and practice. Below I highlight a few suggestions for the authors to consider in revising the article.

1) In the results (Table 2), in the discussion and in the conclusion, there is a significant focus on the differences between children of HIV+ mothers and those of HIV- mothers with the conclusion referring to this work as extending “PMTCT retention research”. If HIV is an important aspect of this research, then it would be useful to highlight this aspect both in the title and in the study objective so that the reader is not surprised when the results are presented.

2) Page 6, line 10: it may be helpful to clarify that cohabitation with the mother refers to mother-child cohabitation. At first reading, it is unclear whether the authors are speaking to the mother’s cohabitation with her own mother (potentially reflecting childcare support).

3) Table 2: the labels for the food security variable may have been switched. If the overall prevalence of food insecurity is 9.6%, then it is odd to have the food insecurity prevalence for children of HIV+ mothers to be 83.5% and 94.3% for those with HIV- mothers.

4) Limitations: About 7% of children (n=40) with the Road to Health book who had at least 19 months of well-care follow up were excluded because the original books had been replaced or were damaged (page 5). Is it possible that this group may differ from the group that had a complete booklet and had they been included would the findings have been different? E.g., could they have lower awareness about the importance of vaccination records?

Minor edits

• Summary of strengths and limitations; page 3, line 15: correct "…but were not measured…"

• Background (page 4, line 7 and line 12): consider using "older mothers" rather than "adult mothers" as adolescents aged 18 and 19 in most settings are legally adults

• Discussion, page 12, line 59. Consider "…mothers who deliver through caesarean section may perceive…"

• Table 2 and 3: label the birth cohort variable categories consistently as 2009 EPI and 2015 EPI, same for food security (no and yes)

**Reviewer**	**2**
**Name**	**Mphasha, Mabitsela**
**Affiliation**	**University of Limpopo, Department of Public Health**
**Date**	**16-Sep-2025**
**COI**	**None**

The study addresses an important and timely topic with clear objectives, but several areas require improvement before it can be considered for publication. The abstract should be revised to present a more balanced account of the findings, giving greater weight to the study's limitations alongside its contributions. The methods section would benefit from more detailed descriptions of the sampling strategy, recruitment process, and analytic framework to enhance transparency and reproducibility. References also need to be updated to include more recent literature from 2021–2024 to ensure the study is grounded in current evidence. The limitations section should be expanded to explicitly address issues of generalisability, contextual constraints, and potential sources of bias. Tables and figures should be simplified and refined to improve clarity, while the overall manuscript would benefit from language and grammar editing to strengthen readability and flow. Finally, the

discussion should integrate findings more cautiously, avoiding interpretations that extend beyond the evidence presented.

Reviewer **3**

Name **Teasdale, Chloe A**

Affiliation **City University of New York (CUNY) Graduate School of Public Health and Health Policy , Department of Epidemiology and Biostatistics**

Date **24-Oct-2025**

COI **None**

This paper addresses an important question and is well written however I have major concerns about the recruitment of the sample and the limitations that introduces which are not well described in the methods and discussion.

Abstract – Overall very clear but not sure what “universal interventions” means in conclusion, recommend clarifying.

Intro – overall very well written, clear and well referenced but in Line 37, not sure what is meant by “factors that promote retention”, seems like the goal of the paper is to describe characteristics associated with retention which may be predictive but not causal, recommend revising language to make clear.

Methods-

It’s not clear why this is called an ‘observational cohort’. A cohort study implies that the study sampled participants before the outcome occurred (either in real time or using records for all eligible children) but it seems clear that this study sampled participants after the outcomes had occurred. I think the authors should change the description of the study to cross sectional study or refrain from calling it an observational cohort (which is not as there was no follow-up of a cohort).

Methods/Results/Discussion concern: approach used to recruit the sample raises some important concerns and limitations that are not described:

1. My understanding is that the researchers recruited children (with caregivers) who attended a clinic visit between March 2017-July 2019 and used RTH cards and caregiver info to document outcomes. More information is needed in the Methods describing the researchers selected and recruited the sample – i.e. how did they identify participants to recruit? For instance, did they try to identify the underlying cohort of eligible children for the study or did they take a convenience sample of children who happened to attend care at the clinics? Also, who did the recruitment, was it nurses or study staff?

2. Sampling children who came to the clinic for care to see whether they had received early life care will very likely oversample children who attended early life visits relative to the entire population of children (which includes children who may have received little or no early life care but were not recruited into the study b/c they still aren't coming for care). I think the authors need to spend more time describing how their sample and results differ from a population-based sample (which this is not) that would have been recruited at birth and followed forward. I understand that is a much more difficult, expensive and time consuming study to do and am not proposing the current study doesn't have value, but more consideration and description of how the findings from this study relate to what the authors likely aimed to do, i.e. describe attendance among all children (not just those currently in care), is needed so the reader can understand who the findings apply to.

3. Related to the above comment, throughout the Methods, Results and Discuss, I think the authors need to make it clear that their findings are limited only to children currently attending care. There may be many children who are no longer in any type of routine healthcare and therefore are not in the sample and it's possible that the findings might be different if those children were included.

4. The same issues around who was excluded and therefore how this study's findings relate to the overall population also need to be considered in regards to the requirement that children had to have complete Road to Health cards. Clearer information (a figure perhaps) explaining how many children >19 months were excluded due lack of RTH info is needed, as well as a more thorough description of how this exclusion may have had an impact on results, are needed.

5. On line 31/32 (page 5) it's not clear what the following means: "of whom 542 had at least 19 months of well-care follow-up records." I'm not sure whether this is describing exclusion of children <19 months of age at the time data collection or whether this means only children with complete records through 18 months were included. The latter would seemingly indicate that the study selected children based on complete outcomes and that almost half of all eligible children were excluded due to this criteria. This needs to be clarified.

6. It's also not clear why the researchers included children up to the age of 9 years of age. This seems like another a potential for significant bias as it will likely oversample children who have remained and been adherence to care, i.e. children coming for visits at older ages are probably more likely to have been brought for more care during infancy and early childhood, compared to children who don't come for care at all. Can the authors explain why they included older children? They might also consider doing a sensitivity analysis to examine whether excluding older children (>3 or >5 years changes their results (for instance).

VERSION 1 - AUTHOR RESPONSE

Thank you for your thoughtful engagement with our manuscript and for proposing valuable suggestions to improve it further. For ease of review, all comments are addressed directly below with a summary of revisions made to the manuscript. All revisions can be traced in the enclosed document with track changes.

Where reviewers raised similar points, we have grouped and addressed these comments together at the beginning of this document. The remaining comments are addressed sequentially by reviewer. Original reviewer comments appear in blue, our responses in black, and summaries of manuscript revisions are presented in boxes.

Comments regarding children excluded from analysis: Both Reviewer 1 and Reviewer 3 raised whether the exclusion of children without available original Road to Health booklets may have biased results, and whether these excluded children may differ systematically from those included:

- Reviewer 1: "(5) Limitations: About 7% of children (n=40) with the Road to Health book who had at least 19 months of well-care follow up were excluded because the original books had been replaced or were damaged (page 5). Is it possible that this group may differ from the group that had a complete booklet and had they been included would the findings have been different? E.g., could they have lower awareness about the importance of vaccination records?"
- Reviewer 3: "(21d) The same issues around who was excluded … a more thorough description of how this exclusion may have had an impact on results, are needed."

Response: To assess whether excluded children differed from those included in this analysis, we conducted a comparison of characteristics between children with available original Road to Health booklets (n=502) and those excluded due to missing or damaged booklets (n=40) (see Supplement 6). Children without available booklets were slightly older at baseline (median 3.1 years vs. 2.3 years; p=0.01) and more likely to have been born under the earlier (2009 EPI) vaccine schedule. This difference is expected, as older children have had their booklets in use for longer, increasing the likelihood of wear, loss, or damage. Road to Health booklets are handled frequently during clinic visits in South Africa, and children actively attending health facilities may be especially prone to booklet deterioration. We also note that excluded children were also more likely to be born with low birthweight; however, exploratory analysis found no association between birthweight and retention. We found no evidence of differences in factors associated with retention in the multivariable analysis (i.e., maternal education, residence, access to the child support grant). While exclusion reduced the sample size slightly, it is unlikely to have introduced bias or influenced the observed associations in the analysis. This is noted in the limitations section of the manuscript:

Lastly, a small proportion of eligible children were excluded due to missing or damaged Road to Health booklets. Compared to those included, excluded children did not differ on factors associated with well-care retention in this study (see Supplement 6). However, children excluded from the analysis were slightly older at the time of data collection, likely because older children are more likely to having lost or damaged

booklets, either due to prolonged use or reduced need for the booklet when they are older. In addition, excluded children were also more likely to be born with low birthweight; however, we found no association between birthweight and retention. *[Page 15, Line 550]*

Supplement 6 Comparison of characteristics of participants with and without Road to Health booklets

	Total (n=542)	Road to Health booklet available (n=502)	Road to Health booklet not available (n=40)	p-value[2]
		Frequency, n (%)		
**Socio-economic & demographic factors**				
**Sex**				
Male	260 (48)	245 (48.8)	15 (37.5)	0.2
Female	282 (52)	257 (51.2)	25 (62.5)	
**Child age at data collection** (years, median [IQR])	2.4 [1.7, 3.3]	2.3 [1.7, 3.2]	3.1 [2.3, 3.7]	0.01
**Birth cohort**				
2009 EPI	245 (45.2)	217 (43.2)	28 (70)	0.001
2015 EPI	297 (54.8)	285 (56.8)	12 (30)	
**Birth order**				
Eldest	507 (93.5)	472 (94)	35 (87.5)	0.1
Second/Third oldest	35 (6.5)	30 (6)	5 (12.5)	
**Residence**				
Urban	384 (70.8)	358 (71.3)	26 (65)	0.4
Rural	158 (29.2)	144 (28.7)	14 (35)	
**Child age at grant receipt**				
None	79 (14.8)	71 (14.3)	8 (20)	0.2
0-4 weeks	99 (18.5)	88 (17.8)	11 (27.5)	
5-14 weeks	69 (12.9)	63 (12.7)	6 (15)	
≥3 months	288 (53.8)	273 (55.2)	15 (37.5)	
**Maternal age at birth[3]** (years, median [IQR])	16.9 [15.8, 18.1]	16.9 [15.8, 18.1]	17.2 [15.8, 18.5]	0.7[4]
**Maternal education (highest grade attended/completed at age of pregnancy)[3]**				
Primary (Grade ≤8)	125 (25.4)	112 (24.4)	13 (38.2)	0.07
Secondary (Grade 9-12)	368 (74.6)	347 (75.6)	21 (61.8)	
**Food Security**				
No	486 (90.3)	48 (9.6)	4 (10)	0.9
Yes	52 (9.7)	450 (90.4)	36 (90)	
**Maternal HIV status[5]**				
Negative	339 (62.5)	319 (63.5)	20 (50)	0.09
Positive	203 (37.5)	183 (36.5)	20 (50)	
**Cohabitation**				
≥1 nights/week	507 (93.5)	470 (93.6)	37 (92.5)	0.8
None	35 (6.5)	32 (6.4)	3 (7.5)	
**Birth outcome**				
**Delivery mode**				
Vaginal delivery	406 (74.9)	375 (74.7)	31 (77.5)	0.7
Caesarean section	136 (25.1)	127 (25.3)	9 (22.5)	
**Birth weight**				
Normal birthweight ≥2500g	456 (85.7)	432 (87.1)	24 (66.7)	0.001
Low birthweight (<2500g)	76 (14.3)	64 (12.9)	12 (33.3)	
**Maternal health services**				
**Number of antenatal care visits attended[3]**				
None	14 (2.8)	12 (2.5)	2 (5.7)	0.5
1-4 appointments	72 (14.2)	68 (14.4)	4 (11.4)	
≥5 appointments	346 (68.2)	324 (68.6)	22 (62.9)	
Don't remember	75 (14.8)	68 (14.4)	7 (20)	
**Trimester at first antenatal care visit[3]**				
First	229 (45.5)	210 (44.9)	19 (54.3)	0.6
Second	220 (43.7)	207 (44.2)	13 (37.1)	
Third/During birth	54 (10.7)	51 (10.9)	3 (8.6)	
**Number of postnatal care visits attended[3]**				
None	173 (34.6)	164 (35.2)	9 (26.5)	0.3
≥1 appointment	327 (65.4)	302 (64.8)	25 (73.5)	

[1]Unconfirmed if these children were HIV-exposed at birth as adolescent mothers may have acquired HIV after child's birth; [2]Obtained using Pearson's chi-squared test; [3]At birth of first order child (n=507); [4]Obtained using Wilcoxon rank-sum test; [5]Unconfirmed if these children were HIV-exposed at birth as adolescent mothers may have acquired HIV after child's birth.

Comments regarding clarification on sampling strategy and recruitment process: Reviewers 2 and 3 both proposed to include more clarity and detail in the Methods section regarding the study's sampling strategy and recruitment process:

- Reviewer 2: "(13) The methods section would benefit from more detailed descriptions of the sampling strategy, recruitment process, and analytic framework to enhance transparency and reproducibility."
- Reviewer 3: "(21a) My understanding is that the researchers recruited children (with caregivers) who attended a clinic visit between March 2017-July 2019 and used RTH cards and caregiver info to document outcomes. More information is needed in the Methods describing the researchers selected and recruited the sample – i.e. how did they identify participants to recruit? For instance, did they try to identify the underlying cohort of eligible children for the study or did they take a convenience sample of children who happened to attend care at the clinics? Also, who did the recruitment, was it nurses or study staff?

Response: In response, we have included more detail in the Methods section to strengthen the transparency and reproducibility of the study methods.

Specifically, we revised the sections titled 'Study design, recruitment and data collection' and 'Sampling' to clearly distinguish between the recruitment process and the sampling strategy. These revisions clarify how adolescent mothers and their children were recruited into the cohort study, and how children were subsequently sampled from the cohort for the analysis in the present study.

As Reviewer 3 requested, we also clarified how participants were identified, whether recruitment was convenience-based or cohort-driven, and who conducted the recruitment. The cohort study first recruited adolescent and young mothers through health facility-based and community-based approaches. Recruitment methods were designed to be demographically diverse and inclusive of adolescent mothers engaged and not engaged in services. Once mothers were enrolled, all children of adolescent and young mothers were eligible for inclusion in the cohort study. As a result of the combined recruitment approach (i.e. health facility-based and community-based), children included in this study represent children of adolescent mothers who have variable engagement with health services. Additional detail has been added to the Methods section to clarify this. As proposed, we also clarified that trained research staff conducted all data collection activities (including recruitment).

To further clarify the **recruitment process and sampling strategy**, we have:

- Renamed the "Study design and data collection" subsection to "Study design, recruitment and data collection" to reflect that this section outlines both how participants were recruited into the cohort as well as the rationale for the recruitment approach. *[Page 5, Line 113]*
- This section now clarifies how health facility- and community-based strategies were used to reduce selection bias toward health service users and to ensure the inclusion of adolescent mothers (and their children) with varying levels of engagement in health services. We also provide justification for this approach

with reference to recent WHO guidance encouraging decentralised, community-based research to enhance representativeness.

… Adolescent and young mothers were recruited using both health facility-based and community-based approaches, including government healthcare facilities providing HIV services, maternity obstetric units, door-to-door recruitment, secondary schools, non-governmental organisation and social service referrals, peer/self-referral, and other community venues (e.g., hair salons, malls, churches) [33]. All children born to eligible mothers were eligible for enrolment in the cohort study, regardless of their cohabitation arrangements with their biological mother. This sampling strategy, implemented across rural and urban settings, improved representativeness and reduced selection bias toward health service users, addressing a common limitation in prior research that relied solely on health facility-based sampling [27,30–32,34]. It enabled inclusion of mothers and children with limited or no health service access and aligns with WHO guidance promoting decentralised, community-based recruitment to enhance representativeness and relevance of research findings [35]. *[Page 5; line 116]*

- Renamed the "Recruitment" subsection to "Sampling" to clarify that this subsection of the Methods explains how children was selected from the cohort for inclusion in this analysis based on data availability and eligibility criteria. [*Page 5; line 139]*

All data collection activities, including the informed consent and assent process, are administered by trained research staff with extensive experience working with vulnerable populations. [Page 8; line 254]

Reviewer #1

In this manuscript, the authors examine the factors that are associated with optimal well-care child visit retention for children of adolescent mothers. The paper is well written and offers valuable insights to inform future research and practice. Below I highlight a few suggestions for the authors to consider in revising the article.

1) In the results (Table 2), in the discussion and in the conclusion, there is a significant focus on the differences between children of HIV+ mothers and those of HIV- mothers with the conclusion referring to this work as extending "PMTCT retention research". If HIV is an important aspect of this research, then it would be useful to highlight this aspect both in the title and in the study objective so that the reader is not surprised when the results are presented.

Response: We have revised the manuscript to better highlight the study's focus on maternal HIV status. Specifically, we have:

- Updated the title to include "*living with and without HIV*" to reflect the relevance of HIV in the study population: *Factors Associated with Retention in Routine Well-Care Visits among Children of Adolescent Mothers living with and without*

HIV: A community-based study in the Eastern Cape, South Africa. [Page 1, Line 2]

- Revised the objective in the abstract: *To describe well-care visit attendance among children of adolescent mothers living with HIV and HIV-negative adolescent mothers and identify factors associated with optimal retention in the well-care visit schedule up to 18 months*. [Page 2, Line 17]
- Updated the conclusion in the abstract: *Universal interventions are needed for retention beyond the neonatal period for children of adolescent mothers living with HIV and HIV-negative adolescent mothers*. [Page 2, Line 41]

2) Page 6, line 10: it may be helpful to clarify that cohabitation with the mother refers to mother-child cohabitation. At first reading, it is unclear whether the authors are speaking to the mother's cohabitation with her own mother (potentially reflecting childcare support).

Response: To clarify this point, the Methods section has been revised to specify that child-mother cohabitation was defined as the child living with their biological mother at least one night per week. We also clarified that data were collected from primary caregivers where *adolescent* mothers were not cohabiting with their children.

- *Data about children's access to health services were collected from primary caregivers where adolescent mothers were not cohabiting with their children. [Page 5, Line 132]*
- *Child-mother cohabitation was defined as the child living with their biological mother at least one night per week. [Page 6, Line 196]*

3) Table 2: the labels for the food security variable may have been switched. If the overall prevalence of food insecurity is 9.6%, then it is odd to have the food insecurity prevalence for children of HIV+ mothers to be 83.5% and 94.3% for those with HIV- mothers.

Response: Thank you for identifying this error, indeed the rows were mistakenly switched. This is now corrected in the revised manuscript.

4) Limitations: About 7% of children (n=40) with the Road to Health book who had at least 19 months of well-care follow up were excluded because the original books had been replaced or were damaged (page 5). Is it possible that this group may differ from the group that had a complete booklet and had they been included would the findings have been different? E.g., could they have lower awareness about the importance of vaccination records?

This point has also been raised by another reviewer. Please see the detailed response above under 'Comments regarding children excluded from analysis' (see page 1).

Minor edits

5) Summary of strengths and limitations; page 3, line 15: correct "...but were not measured..."

6) Background (page 4, line 7 and line 12): consider using "older mothers" rather than "adult mothers" as adolescents aged 18 and 19 in most settings are legally adults
7) Discussion, page 12, line 59. Consider "...mothers who deliver through caesarean section may perceive..."
8) Table 2 and 3: label the birth cohort variable categories consistently as 2009 EPI and 2015 EPI, same for food security (no and yes).

Response: Thank you for your detailed read and identifying these errors, the above suggested edits have been addressed in the revised manuscript.

Reviewer #2

The study addresses an important and timely topic with clear objectives, but several areas require improvement before it can be considered for publication.

9) The abstract should be revised to present a more balanced account of the findings, giving greater weight to the study's limitations alongside its contributions.

Response: Thank you for this suggestion. In line with the journal's author guidelines, the study's strengths and limitations are presented in a standalone "Strengths and limitations of this study" section, which appears alongside the abstract in the final publication. To avoid redundancy and preserve the clarity of the abstract, we propose not duplicating these points within the abstract itself.

10) The methods section would benefit from more detailed descriptions of the sampling strategy, recruitment process, and analytic framework to enhance transparency and reproducibility.

Other reviewers have similarly requested additional information the study's sampling strategy and recruitment process. Please see the detailed response above under 'Comments regarding clarification on sampling strategy and recruitment process' (see page 3).

To further clarify the **analytic framework**, we updated the Conceptual Framework and Outcome Measure subsections in the Methods to define each variable in more detail and explaining the rationale for their inclusion. This includes clearer definitions of maternal HIV status, food security, caregiving support, and other variables within each level of the framework. We also provide additional justification for selecting eight WHO-aligned visits and explain how the binary outcome of "optimal retention" was constructed.

... Level 1 includes socio-economic factors (i.e., food security, maternal level of education) and demographic characteristics (i.e., child sex, child's birth cohort, urban/rural residence, maternal age at birth, and maternal HIV status). Birth cohort was included to account for the 2015 update to South Africa's Expanded Programme on Immunisation (EPI), which added vaccinations to two existing well-care visits [48]. This may have prompted more consistent attendance among children born after the

vaccination schedule change. Children were categorised as being born before (i.e. EPI 2009) or after (i.e. EPI 2015) the programmatic update. Food security was defined as experiencing at least one day without enough food at home in the past week and was included as a proxy for household income [38]. Maternal HIV status was recorded only at the time of data collection and verified using self-report and Road to Health booklet data. While this may not reflect whether a child was HIV-exposed in utero or during the first 18 months of life, it was included as a variable to capture differences in the lived experiences of children and families affected by HIV. Level 2 captures maternal health behaviours prior to and during childbirth, including antenatal care attendance and delivery mode. Attendance at antenatal care is a known predictor of complete and timely childhood vaccination and was measured as the number of antenatal visits reported by the mother [35,36]. Caesarean section delivery may influence well-care visit attendance, either by increased perceived need for postnatal care or by creating mobility challenges during recovery. In South Africa, caesarean section is not a recommended practice without valid indication [39]. Caesarean section delivery was identified using records in the Road to Health booklet and supplemented by maternal self-report where missing. Level 3 contains two groups of enabling factors: support-related factors (i.e., access to caregiving support, child and mother cohabitation, access to child support grant and age at which the child's mother returned to school) and health services factors (i.e., healthcare worker attitudes, waiting time at the clinic and distance to the clinic). Child-mother cohabitation was defined as the child living with their biological mother at least one night per week. Access to caregiving support was defined as mothers receiving help with childcare, household tasks, or buying essentials for the child at least once every two weeks. Healthcare worker attitudes were captured through reports of whether the mother was shouted at during childbirth. [Page 6; line 176]

.... First, we created a variable for attendance for eight well-care visits (i.e., 3–6 day visit, 6-, 10- and 14-week visits, and 6-, 9-, 12-, and 18-month visits) aligning with WHO guidance [4] and the South African schedule [14] (Table 1). The decision to focus on these eight WHO-aligned visits was based on methodological and conceptual considerations. The aim was to assess visit constancy and schedule completion, not visit frequency. Additional visits in the South African schedule between WHO-recommended visits are primarily for growth monitoring and do not introduce new interventions (Table 1) and since children are typically caught up on missed care at subsequent visits, any one visit within the interval was considered sufficient to reflect receipt of well-care interventions in line with children's age. Finally, aligning with the WHO schedule supports comparability with studies beyond South Africa. Attendance was determined by whether a well-care or vaccination date was recorded within specified age thresholds (Table 1), ensuring no overlap between consecutive visits while allowing flexibility in scheduling and preventing double-counting [41,42]. For the first four visits, stricter thresholds aligned with precise age recommendations, counting attendance if a visit occurred one week before or up to three weeks after the recommended age (e.g., 5–8 weeks for the 6-week visit). From 6 months onward, broader thresholds were used to reflect typical scheduling practices, allowing attendance within one month after the recommended age (e.g., 25–33 weeks for the 6-month visit). [Page 6; line 218]

In addition, we revised the Statistical Analysis subsection of the Methods to include more detail and improve clarity about the analytical approach used. Specifically, we clearly describe how the multivariable logistic regression analysis followed a hierarchical modelling approach, guided by the conceptual framework. This analysis followed a hierarchical modelling approach which involves grouping variables by level (based on the conceptual framework) and adjusting for all variables at group level (Victora et al., 1997). This allowed us to examine the association between factors more proximal to the outcome while adjusting for distal factors, which are all treated as potential confounders. Specifically, level 1 variables were treated as confounders for level 2 variables (i.e., Model 2), and levels 1 and 2 were treated as confounders for level 3a and 3b variables (i.e., Model 3a and 3b), respectively. In model 1, all level 1 variables were considered as potential confounders of the relationship between other level 1 variables and retention. We have revised the description of the statistical analysis to support reproducibility and clarify how variables were interpreted in each model.

... In Model 1, we examined the association of level 1 variables (socio-economic and demographic characteristics) with well-care visit retention, treating level 1 variables as potential confounders of the relationship between other level 1 variables and retention. Model 2 added level 2 variables (maternal health-seeking behaviours), adjusting for level 1 factors considered as confounders for the association between maternal health-seeking behaviours and retention. Finally, Model 3a and 3b estimated the effects of level 3a (support-related factors) and 3b (health services factors) variables, respectively, adjusting for level 1 and 2 variables, which were retained as confounders in line with the hierarchical modelling approach. [Page 8; line 286]

11) References also need to be updated to include more recent literature from 2021–2024 to ensure the study is grounded in current evidence.

Response: We appreciate the suggestion to ensure that the manuscript is grounded in recent literature. The reference list has been reviewed and currently includes 25 references relevant to the use of child health services delivered at primary care facilities in South Africa (e.g., vaccination, growth monitoring, PMTCT), of which 7 were published between 2021 and 2024. Additionally, in response to this comment, we incorporated one additional reference (Javadi et al. 2023) of a review on adolescent girls' postnatal care experiences. This was identified following a recent literature review conducted by the first author aiming to identify and summarise studies that (1) measure coverage and utilisation of well-care services and (2) examine factors influencing utilisation of well-care services quantitatively and qualitatively.

▪ *Additionally, increased interaction with healthcare workers during and after a caesarean section may support continued retention beyond the postpartum period. Recent evidence shows that adolescent mothers frequently experience judgement, stigma, and limited emotional support during postnatal care, which can undermine engagement [22]. Mothers who have undergone surgery are likely to receive more attention from healthcare workers by receiving more*

postnatal guidance and discharge counselling than mothers giving birth by normal vaginal delivery. [Page 14; line 459]

- *Children of mothers who returned to school in this study may be able to maintain access to well-care visits due to availability of caregiving support which facilitate their children's access to health services [65]. Kinship networks are central to sustaining postnatal care engagement among adolescent mothers [22]. Although informal childcare is common in South Africa [66], formal childcare services remain crucial for supporting young mothers and improving outcomes for their children [67]. [Page 14; line 478]*

12) The limitations section should be expanded to explicitly address issues of generalisability, contextual constraints, and potential sources of bias.

Response: We have expanded the limitations section to address issues of generalisability, contextual constraints, and potential sources of bias, including misclassification and selection bias. We acknowledge that several variables (e.g., maternal return to school, child-mother cohabitation, residence) were measured cross-sectionally while the outcome was assessed longitudinally. This temporal mismatch could lead to non-differential misclassification, potentially biasing associations toward the null. However, we note in the revised limitations section that exploratory analyses among children who had completed the 18-month observation period prior to measurement of maternal return to school found no meaningful association between this variable and retention, suggesting limited impact on the findings. In addition, we also added discussion of the potential for differential misclassification due to response bias for healthcare worker attitudes, which may have led mothers to underreport harsh treatment. This could underestimate the effect of poor service quality on retention.

To state more clearly the generalisability of our findings, we clarify that the sample includes adolescent mothers and their children recruited using both health facility- and community-based strategies across urban and rural areas. While findings may not be transferrable to other settings where health systems or norms differ, the representativeness of the sample for adolescent mothers in this district, and similarities in barriers to access faced in other provinces, support the relevance of these findings for similar contexts.

Additionally, we address selection bias related to the exclusion of children with missing or damaged Road to Health booklets. While this reduced the sample size, descriptive comparisons showed that excluded children did not differ from included children on factors associated with retention – supporting the validity of the findings.

These revisions are reflected in the updated 'Strengths and Limitations' section of the manuscript.

While the temporal direction of exposures measured before childbirth (such as maternal health-seeking behaviour and education) provides evidence for potential causal pathways to retention, other risk factors were measured cross-sectionally, limiting our ability to explore causal links between support-related and healthcare service factors and the outcome. Unmeasured factors, such as maternal knowledge about well-care visits and vaccine stock-outs, could further explain variations in

retention. Potential response bias may have led to an underestimation of the impact of service quality on retention, as mothers who experienced harsh treatment from healthcare workers during childbirth may have been less willing to disclose it. Maternal return to school was measured only at baseline, which may have led to non-differential misclassification and underestimation of the number of mothers who returned to school during the 18-month observation period. However, exploratory analyses among children over 19 months old at baseline did not show an association between maternal return to school and well-care visit retention, suggesting minimal impact on the findings. Similarly, potential non-differential misclassification of residence, measured cross-sectionally and possibly affected by population mobility, is unlikely to have meaningfully influenced the results. Lastly, a small proportion of eligible children were excluded due to missing or damaged Road to Health booklets. Compared to those included, excluded children did not differ on factors associated with well-care retention in this study (see Supplement 6). However, children excluded from the analysis were slightly older at the time of data collection, likely because older children are more likely to having lost or damaged booklets, either due to prolonged use or reduced need for the booklet when they are older. In addition, excluded children were also more likely to be born with low birthweight; however, exploratory analysis found no association between birthweight and retention. [Page 15; line 535]

13) Tables and figures should be simplified and refined to improve clarity, while the overall manuscript would benefit from language and grammar editing to strengthen readability and flow.

Response: Thank you for this feedback. We have carefully reviewed the manuscript, including all tables and figures, and ensured that the presentation is as clear and accessible as possible. In this process, we made minor edits to improve grammar and readability to ensure accessibility of the manuscript.

14) Finally, the discussion should integrate findings more cautiously, avoiding interpretations that extend beyond the evidence presented.

Response: We have reviewed the Discussion and made several refinements to strengthen caution in interpretation of the findings of the present study. Specifically, we have (1) included minor edits to the reporting of the results to support interpretability and (2) described results with appropriate attention to the strength of statistical evidence (e.g., using "weak evidence" or "no evidence"). We have more clearly specified the population to which the findings apply and included more detail in the limitations section of the discussion.

Reviewer #3

This paper addresses an important question and is well written however I have major concerns about the recruitment of the sample and the limitations that introduces which are not well described in the methods and discussion.

15) Abstract – Overall very clear but not sure what "universal interventions" means in conclusion, recommend clarifying.

Response: The terms universal, selective and indicated interventions are commonly used lexicon to refer to whether public health interventions should target populations universally (i.e. all), selectively (i.e. groups with increased risk), or indicated (i.e. individuals with existing need). Given this term is widely used in global and public health research, we would prefer to keep the term and have elaborated further that this refers to both children of adolescent mothers living with HIV and HIV-negative adolescent mother (i.e. interventions should be universal and not target by maternal HIV status).

Universal interventions are needed for retention beyond the neonatal period for children of adolescent mothers living with HIV and HIV-negative adolescent mothers. [Page 2; line 40]

16) Intro – overall very well written, clear and well referenced but in Line 37, not sure what is meant by "factors that promote retention", seems like the goal of the paper is to describe characteristics associated with retention which may be predictive but not causal, recommend revising language to make clear.

Response: thank you for highlighting this nuance. Indeed, this study examined factors associated with optimal retention and did not examine any interventions that may increase retention among those with low-attendance and can therefore not infer how to 'promote' retention. We amended several sentences in the Background and Discussion to ensure the word choice accurately reflects the intended objectives and how findings should be interpreted.

- *Little is known about factors that are associated with ~~promote~~ retention in well-care visits beyond the neonatal period, particularly for children without perinatal HIV exposure and those born to adolescent mothers. [Page 4; line 84]*
- *Identifying factors that are associated with retention is crucial to support ~~promote~~ access to essential child health services, particularly for children of adolescent mothers, who face increased risks of adverse health and developmental outcomes. [Page 4; line 103]*
- *Reinforcing the importance of well-care visits and improving service quality may support ~~promote~~ retention. [Page 14; line 508]*

17) Methods- It's not clear why this is called an 'observational cohort'. A cohort study implies that the study sampled participants before the outcome occurred (either in real time or using records for all eligible children) but it seems clear that this study sampled participants after the outcomes had occurred. I think the authors should change the description of the study to cross sectional study or refrain from calling it an observational cohort (which is not as there was no follow-up of a cohort).

Response: This is an important clarification, and we agree the study design should be clearer. While the cohort from which data are drawn is a prospective longitudinal study (February 2017 - June 2025), the analysis conducted for this study used (1) cross-sectional data collected at baseline (February 2017 - July 2019) (i.e. self-reported data

collected from surveys about children's access to health services) and (2) data extracted from children's Road to Health booklets throughout the cohort (i.e. Road to Health booklet data was used to retrospectively assess attendance during their first 18 months (birth – 18 months old) of the well-care visit schedule). Relevant sections have been updated to reflect this more clearly.

- ***Design, setting, participants:*** *Cross-sectional data were used from a community-based observational cohort study of adolescent mothers (10-19 years; n=481) and their children (≥19 months old; n=502) in the Eastern Cape, South Africa. [Page 2; line 21]*
- *Cross-sectional data (2017 – 2019) about children's access to health services were collected from primary caregivers where adolescent mothers were not cohabiting with their children. [Page 5; line 131]*

18) Methods/Results/Discussion concern: approach used to recruit the sample raises some important concerns and limitations that are not described:

Response: We appreciate your concern about the potential for selection bias related to recruitment procedures and the risk of oversampling children engaged in care. However, after carefully reviewing the comments, we infer that there may be a misunderstanding about the study's design and methods which directly mitigate for these issues.

To clarify, children were enrolled through their mothers and children were *not* recruited at clinics and therefore their inclusion in the study was not based on clinic attendance. Rather, adolescent and young mothers (aged 10–24) were first recruited into the observational cohort study using both health facility- and community-based approaches. These strategies were designed to include mothers with variable access to health services and to reduce bias toward mothers (and their children) with current access to services. All children born to enrolled adolescent mothers were then included in the cohort.

Children were not selected based on attendance to health facilities. Instead, we identified a sub-sample from the full cohort for this analysis based on age eligibility (≥19 months at baseline) and availability of Road to Health booklets, which provided historical data on their well-care visit attendance from birth to 18 months. We only included children who were at least 19 months old when we last captured their Road to Health booklet data to allow us to observe their well-care visit attendance at facilities up to the 18-month visit and allowing 4 weeks of additional observations after the 18-month visit – hence, 19 months.

These procedures are described in the Methods, which we have further clarified in the revised manuscript to avoid this confusion. We have also added clearer discussion of how this cohort study differs from a population-based birth cohort and acknowledged limitations in generalisability in the Discussion. We hope this address concerns about how and when children entered the study and how well-care attendance outcomes were measured.

(a) My understanding is that the researchers recruited children (with caregivers) who attended a clinic visit between March 2017-July 2019 and used RTH cards and caregiver info to document outcomes. More information is needed in the Methods

describing the researchers selected and recruited the sample – i.e. how did they identify participants to recruit? For instance, did they try to identify the underlying cohort of eligible children for the study or did they take a convenience sample of children who happened to attend care at the clinics? Also, who did the recruitment, was it nurses or study staff?

This point has also been raised by another reviewer. Please see the detailed response above under 'Comments regarding clarification on sampling strategy and recruitment process' (see page 3).

Comments regarding selection bias and representativeness of the sample:

(b) Sampling children who came to the clinic for care to see whether they had received early life care will very likely oversample children who attended early life visits relative to the entire population of children (which includes children who may have received little or no early life care but were not recruited into the study b/c they still aren't coming for care). I think the authors need to spend more time describing how their sample and results differ from a population-based sample (which this is not) that would have been recruited at birth and followed forward. I understand that is a much more difficult, expensive and time consuming study to do and am not proposing the current study doesn't have value, but more consideration and description of how the findings from this study relate to what the authors likely aimed to do, i.e. describe attendance among all children (not just those currently in care), is needed so the reader can understand who the findings apply to.

(c) Related to the above comment, throughout the Methods, Results and Discuss, I think the authors need to make it clear that their findings are limited only to children currently attending care. There may be many children who are no longer in any type of routine healthcare and therefore are not in the sample and it's possible that the findings might be different if those children were included.

Response: Thank you for making this important remark. We agree that clarifying the representativeness of the sample is important for interpreting findings to ensure readers appropriately infer the generalisability of the findings. We would like to emphasise that the findings are not limited only to children currently attending care. Recruitment was designed to include adolescent mothers and their children with varying levels of engagement with health services. Mothers were recruited through both health facility-based and community-based strategies, and all their children were eligible for enrolment, regardless of their access to or use of health services. Children were not recruited at clinics based on attendance but rather through their mothers. This strategy was used to mitigate selection bias toward children with current health service access and ensure a more representative sample of adolescent mother-child dyads.

To further clarify this, we have revised the Methods section to describe the combined recruitment strategy in more detail. Additionally, we have added a statement to the Discussion to make the representativeness of the sample and the generalisability of findings more explicit. Specifically, we explain that findings from this study are generalisable to populations of children of adolescent mothers (10-19 years) living in

peri-urban and rural areas in the Eastern Cape, including children of adolescent mothers living with HIV who may not have existing access to health services. We also acknowledge that these findings may not be transferable to other provinces in South Africa, where health service infrastructure and norms around adolescent pregnancy, motherhood, and kinship networks may differ. Nonetheless, these findings provide valuable insights that may be applicable in similar contexts.

Finally, using health facility and community-based recruitment reduced selection bias and improved the generalisability of findings, particularly for children of adolescent mothers in peri-urban and rural areas, who may not have regular access to health services. Findings are generalisable to children of adolescent mothers (10–19 years old) living in peri-urban and rural areas in the Eastern Cape, including those affected by HIV. While generalisability to other provinces may be limited due to differences in health service infrastructure and norms, these findings offer valuable insights for similar contexts across South Africa where adolescent mothers face comparable barriers to access. [Page 15; line 527]

Comments regarding sampling:

(d) The same issues around who was excluded and therefore how this study's findings relate to the overall population also need to be considered in regards to the requirement that children had to have complete Road to Health cards. Clearer information (a figure perhaps) explaining how many children >19 months were excluded due lack of RTH info is needed, as well as a more thorough description of how this exclusion may have had an impact on results, are needed.

(e) On line 31/32 (page 5) it's not clear what the following means: "of whom 542 had at least 19 months of well-care follow-up records." I'm not sure whether this is describing exclusion of children <19 months of age at the time data collection or whether this means only children with complete records through 18 months were included. The latter would seemingly indicate that the study selected children based on complete outcomes and that almost half of all eligible children were excluded due to this criteria. This needs to be clarified.

Response: Thank you for these comments. To clarify, there was no requirement for children to have complete records in their Road to Health booklets - only that their original booklet was available for data extraction. Eligibility for inclusion in this analysis was based solely on children having reached at least 19 months of age at the time their booklet was photographed, enabling us to observe whether they attended the 18-month well-care visit within a four-week window. This approach ensured a consistent follow-up period across all included children, rather than selection based on complete outcomes.

As described in the Methods and illustrated in the sampling flowchart (Supplement 2), of the 1154 children enrolled in the cohort study. Only children born to mothers ≤19 years old and who were born before May 2018 were eligible to be included in the analysis as these children were expected to have attended their 18-month visit by December 2019, allowing four weeks to observe attendance (leaving n=998). This was to ensure that baseline survey data coincided with the period of well-care visit attendance recorded in booklets. Of these, 70 children did not have a Road to Health booklet (leaving n=928), and of these, 542 were at least 19 months old at baseline and therefore eligible

for inclusion based on available follow-up time. Of the 542, 12 children had booklets available, but these were not their original booklets and therefore historical well-care visit data was missing, and 28 children had booklets that were too damaged or had missing pages. Concerns regarding the exclusion of children without Road to Health booklets has also been raised by another reviewer. Please see the detailed response above under 'Comments regarding children excluded from analysis' (see page 1).

Finally, we have made minor edits to the manuscript to clarify that children were not selected based on the completeness of their well-care visit attendance outcomes. Rather, children were only included in this analysis if they had reached 19 months of age at the time of data collection to allow us to measure whether they had attended the 18-month well-care visit within 4 weeks of the recommended age. This does not imply selection based on complete outcomes, but rather eligibility based on sufficient age for follow-up. We have rephrased this section of the manuscript as follows:

> *Complete survey data and original Road to Health booklets were available for 928 children, of whom 542 were at least 19 months old at data collection and had sufficient follow-up well-care follow-up records. Of these, 12 non-original booklets were excluded (i.e., data were missing because the original booklets had been replaced), and 28 were excluded due to extensive damage or missing pages. As a result, 502 children were included in the analysis. [Page 5; line 147]*

(f) It's also not clear why the researchers included children up to the age of 9 years of age. This seems like another a potential for significant bias as it will likely oversample children who have remained and been adherence to care, i.e. children coming for visits at older ages are probably more likely to have been brought for more care during infancy and early childhood, compared to children who don't come for care at all. Can the authors explain why they included older children? They might also consider doing a sensitivity analysis to examine whether excluding older children (>3 or >5 years changes their results (for instance).

Response: As described in the revised Methods section, all children were eligible for inclusion in the cohort based on their mother's eligibility: *"Adolescent and young mothers (10-24 years old) (n=1044) and their children (n=1154) living in the Eastern Cape, South Africa, were recruited into an observational cohort study (March 2017 – July 2019) [33]. Adolescent and young mothers were recruited using both health facility-based and community-based approaches.... All children born to eligible mothers were eligible for enrolment in the cohort study, regardless of their cohabitation arrangements with their biological mother."* [Page 5; line 114]. This resulted in a cohort of children ranging in age from 3 weeks to 9 years at baseline. Recruitment methods were designed to be diverse and inclusive, drawing from both health facilities and community-based approaches to reduce selection bias toward health service users.

For this specific analysis of well-care visit retention up to 18 months, we only included children who were at least 19 months old at the time their Road to Health booklets were photographed. This ensured that the full 18-month period of well-care visits could be observed, regardless of how old the child was at recruitment. Children were not excluded based on their age, as their records allowed us to retrospectively

assess attendance during their first 18 months of the well-care visit schedule. Children who had not yet reached 19 months at the time their Road to Heath data was captured were excluded because they were too young to retrospectively assess attendance during their first 18 months of the well-care visit schedule.

Indeed, health services evolve over time so older children in the sample may have had different experiences compared to younger children. To account for this variation in health services and programming, multivariable analyses adjusted for birth cohort. This variable was defined based on whether children were born before or after the 2015 revision of the South African Expanded Programme on Immunisation (EPI), which additionally integrated vaccinations at two well-care visits. Given that birth cohort was already included in the multivariable model to account for age and programme differences, we maintain that a separate sensitivity analysis excluding older children would not offer additional insights into retention. For clarity, the rationale for including the birth cohort variable is now included in the Methods.

Birth cohort was included to account for the 2015 update to South Africa's Expanded Programme on Immunisation (EPI), which added vaccinations to two existing well-care visits [48]. This may have prompted more consistent attendance among children born after the vaccination schedule change. Children were categorised as being born before (i.e. EPI 2009) or after (i.e. EPI 2015) the programmatic update. *[Page 6; line 176]*.

VERSION 2 - REVIEW

Reviewer 3

Name Teasdale, Chloe A

Affiliation City University of New York (CUNY) Graduate School of Public Health and Health Policy , Department of Epidemiology and Biostatistics

Date 05-Dec-2025

COI

Thank you for your throughout consideration of the comments and the clear presentation of responses and revisions.
